# Distinct smell and taste disorder phenotype of post-acute COVID-19 sequelae

**DOI:** 10.1007/s00405-023-08163-x

**Published:** 2023-09-05

**Authors:** Verena Rass, Piotr Tymoszuk, Sabina Sahanic, Beatrice Heim, Dietmar Ausserhofer, Anna Lindner, Mario Kofler, Philipp Mahlknecht, Anna Boehm, Katharina Hüfner, Alex Pizzini, Thomas Sonnweber, Katharina Kurz, Bernhard Pfeifer, Stefan Kiechl, Marina Peball, Philipp Kindl, Lauma Putnina, Elena Fava, Atbin Djamshidian, Andreas Huber, Christian J. Wiedermann, Barbara Sperner-Unterweger, Ewald Wöll, Ronny Beer, Alois Josef Schiefecker, Rosa Bellmann-Weiler, Herbert Bachler, Ivan Tancevski, Bettina Pfausler, Giuliano Piccoliori, Klaus Seppi, Günter Weiss, Judith Löffler-Ragg, Raimund Helbok

**Affiliations:** 1 Department of Neurology, Medical University of Innsbruck, Innsbruck, Austria; 2 Data Analytics as a Service Tirol, Innsbruck, Austria; 3 Department of Internal Medicine II, Medical University of Innsbruck, Innsbruck, Austria; 4 Institute of General Practice and Public Health, Claudiana College of Health Professions, Bolzano, Italy; 5 Department of Psychiatry, Psychotherapy, Psychosomatics and Medical Psychology, University Hospital for Psychiatry II, Medical University of Innsbruck, Innsbruck, Austria; 6 Tyrolean Federal Institute for Integrated Care, Innsbruck, Austria; 7 Division for Health Networking and Telehealth, Biomedical Informatics and Mechatronics, UMIT, Hall in Tyrol, Austria; 8 Department of Internal Medicine, St. Vinzenz Hospital, Zams, Austria; 9 Institute of General Medicine, Medical University of Innsbruck, Innsbruck, Austria; 10 Department of Neurology, Johannes Kepler University, Linz, Austria

**Keywords:** Olfactory dysfunction, Smell and taste disorder, Long COVID, Post-COVID-19 condition, Mental health, Quality of life

## Abstract

**Purpose:**

Olfactory dysfunction (OD) commonly accompanies coronavirus disease 2019 (COVID-19). We investigated the kinetics of OD resolution following SARS-CoV-2 infection (wild-type and alpha variant) and its impact on quality of life, physical and mental health.

**Methods:**

OD prevalence was assessed in an ambulatory COVID-19 survey (n = 906, ≥ 90 days follow-up) and an observational cohort of ambulatory and hospitalized individuals (n = 108, 360 days follow-up). Co-occurrence of OD with other symptoms and effects on quality of life, physical and mental health were analyzed by multi-dimensional scaling, association rule mining and semi-supervised clustering.

**Results:**

Both in the ambulatory COVID-19 survey study (72%) and the observational ambulatory and hospitalized cohort (41%) self-reported OD was frequent during acute COVID-19. Recovery from self-reported OD was slow (survey: median 28 days, observational cohort: 90 days). By clustering of the survey data, we identified a predominantly young, female, comorbidity-free group of convalescents with persistent OD and taste disorders (median recovery: 90 days) but low frequency of post-acute fatigue, respiratory or neurocognitive symptoms. This smell and taste disorder cluster was characterized by a high rating of physical performance, mental health, and quality of life as compared with convalescents affected by prolonged fatigue or neurocognitive complaints.

**Conclusion:**

Our results underline the heterogeneity of post-acute COVID-19 sequelae calling for tailored management strategies. The persistent smell and taste disorder phenotype is characterized by good clinical, physical, and mental recovery and may pose a minor challenge for public health.

**Study registration:**

ClinicalTrials.gov: NCT04661462 (survey study), NCT04416100 (observational cohort).

**Supplementary Information:**

The online version contains supplementary material available at 10.1007/s00405-023-08163-x.

## Introduction

Coronavirus disease 2019 (COVID-19) manifests with various respiratory, neurological, neurocognitive and cardiopulmonary symptoms [1–4]⁠. A considerable number of COVID-19 patients suffers from persistent symptoms [2, 4–6]⁠. In a WHO consensus paper, the ‘post-COVID-19 condition’ was defined as any symptoms present for at least 8 weeks at three months after clinical onset [7]. However, this definition does not address the character of persistent symptoms or their burden on daily functioning, quality of life and mental health.

Smell disorder or olfactory dysfunction (OD) affects up to 48% of patients during acute infection with the wild type, alpha and delta SARS-CoV-2 virus variants [8–10]⁠. OD was in turn significantly less common by 80% in the acute omicron SARS-CoV-2 variant infection as compared with the wild type pathogen [9]⁠. Such COVID-19-related OD may result from an injury of upper respiratory epithelial cells or neurons of the olfactory mucosa, olfactory bulb, primary olfactory cortex or secondary projection areas [11, 12]⁠. Although literature suggests resolution of OD within 2–3 weeks in most patients [13–16]⁠, it may persist for at least 6 months in 5–11% of patients [5, 14–21]⁠. Consequently, OD represents a common and important post-acute sequelae, with a disabling character for certain patients due to its effect on daily functioning and professional activity [20, 22–24]⁠. Hence, it is crucial to identify patients at risk of persistent OD who may profit from targeted therapy such as olfactory training [25–27]⁠.

Herein, we investigated clinical and psychosocial recovery in ambulatory and hospitalized COVID-19 patients with a particular focus on OD recovery pace, co-occurrence with other persistent symptoms and effects on quality of life, physical performance, and mental health. We therefore re-analyzed our previously published bi-national survey of non-hospitalized COVID-19 patients (Health after COVID-19 in Tyrol) [1, 3]⁠ and a multi-center observational cohort including both ambulatory and hospitalized patients (CovILD) [2, 5, 28]⁠ recruited during outbreaks of wild type and alpha variant of the SARS-CoV-2 pathogen with association mining and clustering algorithms.

## Methods

### Study design and approval

The ‘Health after COVID-19 in Tyrol’ online survey (ClinicalTrials.gov: NCT04661462), further referred to as ‘survey study’, encompassed two cohorts of adult ambulatory, non-hospitalized individuals with laboratory-confirmed SARS-CoV-2 infection independently recruited in western Austria (AT, Tyrol province) and northern Italy (IT, South Tyrol province) [1]⁠. The survey study was conducted during outbreaks of the wild-type and alpha variant of SARS-CoV-2 (30th September 2020–5th July 2021). COVID-19 convalescents were invited to participate by a public media call (AT and IT) or by general practitioners (IT). The survey inclusion criteria were PCR- or seropositivity-confirmed SARS-CoV-2 infection, residency in the study regions and adult age (AT: ≥ 16, IT ≥ 18 years). The survey exclusion criterion was COVID-19-related hospitalization [1, 29]⁠. The participants with symptomatic COVID-19 and ≥ 90 days between diagnosis and survey completion were included in the analysis (AT: n = 479, IT: n = 427, Supplementary Fig. S1).

The CovILD observational cohort (NCT04416100), further referred to as ‘CovILD cohort’, included COVID-19 outpatients and inpatients recruited at the Department of Internal Medicine II at the Medical University of Innsbruck, St. Vinzenz Hospital in Zams and Karl Landsteiner Rehabilitation Facility in Muenster (all in Austria) during the wild-type SARS-CoV-2 outbreak (March–June 2020) [2, 5, 28, 29]⁠. The participants who had completed all scheduled follow-ups (60-, 100-, 180- and 360-day after diagnosis) were included in the analysis (n = 108, Supplementary Fig. S1).

The studies were conducted in accordance with the Declaration of Helsinki and European Data Policy. All participants gave written informed consent to participate. The study protocols were approved by the ethics committees of the Medical University of Innsbruck (survey study, AT, approval number: 1257/2020, CovILD cohort: 1103/2020) and of the Autonomous Province of Bolzano—South Tyrol (survey study, IT: 0150701).

### Procedures and study variables

In the survey study, participants assigned 42 self-reported symptoms to duration intervals (absent: re-coded as 0 days, 1–3 days: 3 days, up to 1 week: 7 days, up to 2 weeks: 14 days, up to 4 weeks: 28 days, up to or greater than 3 months: 3 months). Acute symptoms were defined as complaints present in the first 14 days after clinical onset. Self-perceived complete recovery, need for rehabilitation and new drugs following COVID-19 were surveyed as single yes/no items. Physical performance loss following COVID-19 was rated with a 0–100% scale [3]⁠. Quality of life and overall mental health impairment (4-item Likert scale each) [3]⁠, anxiety and depression (Patient Health Questionnaire, PHQ-4) [30]⁠ and psychosocial stress (7 item PHQ stress module) [31]⁠ were assessed at the time of survey completion.

In the CovILD cohort, 8 self-reported symptoms (reduced physical performance, OD, dyspnea, sleep problems, cough, fever, night sweating, gastrointestinal symptoms) were recorded at each follow-up. Acute symptoms were assessed retrospectively at the 60-day follow-up [2, 5]⁠. Objective OD was evaluated with the 16-item Sniffin’ Sticks Identification test (Burghart Medizintechnik, Wedel, Germany) at the 100- (n = 95) and 360-day follow-up (n = 63). The nasal chemosensory performance was investigated using pen-like odor-dispensing devices for odor identification of 16 common odorants (multiple forced‐choice from four verbal items per test odorant). Hyposmia was defined for < 13 correctly identified odorants, as per manufacturer criteria [32–36]⁠.

Study variables are listed in Supplementary Table S1 (survey study) and S2 (CovILD cohort).

### Analysis endpoints

The primary analysis endpoint for the ambulatory survey study was the frequency of self-reported OD up to three months after clinical onset of COVID-19. The primary analysis endpoint for the CovILD cohort was the frequency of self-reported OD up to 1 year after after COVID-19 diagnosis.

The secondary analysis endpoint for the CovILD cohort was the comparison of rates of subjective and objective OD. The secondary analysis endpoint for the survey study were co-occurrence of self-reported OD with other symptoms and impact of self-reported OD on self-perceived recovery, physical performance, quality of life and mental health.

### Statistical analysis

Data analysis was conducted with R, version 4.2.3. Numeric variables were presented as medians with interquartile ranges and ranges. Categorical variables are presented as percentages and counts within the complete observation set. Statistical significance was investigated by Mann–Whitney U or paired Wilcoxon test with r effect size statistic, Kruskal–Wallis test with η^2^ effect size statistic, χ^2^ test with Cramer’s V effect size statistic, Cochran Q test with Kendall’s W effect size statistic or McNemar test with Cohen’s g effect size statistic. Subjective and objective OD were compared with Cohen’s κ statistic. Two-dimensional mapping of simple matching distances between symptoms was done by multi-dimensional scaling. Apriori association rule analysis was conducted with *arules* package [37]. Data sets of symptom-specific recovery times in the AT and IT cohorts of the survey study demonstrated good clustering tendency assessed by Hopkins statistic (H; H = 0: uniform distribution, H = 1 highly clustered data; AT: H = 0.80, IT: H = 0.79). Sizes of the AT (n = 479) and IT cohort (n = 427) were sufficient for reliable clustering analysis as investigated by clustering tendencies of random subsets of the pooled AT/IT recovery time data set (minimal cohort size n = 400, Supplementary Fig. S2). Clustering of the training AT cohort by symptom-specific recovery times was done with the PAM algorithm (partitioning around medoids, Euclidean distance) [38]⁠. Assignment of the test IT cohort participants to the clusters was accomplished with the inverse distance-weighted 7-nearest neighbors classifier [39]⁠. P values were corrected for multiple testing with Benjamini–Hochberg method. For details of the statistical analysis, see Supplementary Methods.

## Results

### Study cohorts

Out of 3140 survey study respondents [1]⁠, 906 non-hospitalized individuals with symptomatic COVID-19 and ≥ 90 days after diagnosis were analyzed. Those individuals were grouped in two cohorts independently recruited in western Austria (AT, n = 479) and northern Italy (IT, n = 427), respectively (Supplementary Fig. S1). The median follow-up time ranged from 140 (IT) to 180 days (AT). The cohorts consisted primarily of working-age individuals (AT: median 43 [IQR: 32–53]; IT: 45 [34–54] years) and females were over-represented (AT: 67%, IT: 70%). Almost half of participants had at least one comorbidity (AT: 49%, IT: 43%), with  hay fever/allergy, arterial hypertension, and obesity (body mass index > 30 kg/m^2^) as leading conditions (Table 1). AT cohort was characterized by a significantly longer follow-up time. Most AT cohort participants were infected during the spring 2020 outbreak (AT: 59%, IT: 30%), whereas IT cohort individuals were infected predominantly during the summer/fall 2020 SARS-CoV-2 waves (AT: 40%, IT: 69%). The IT collective was also characterized by a significantly lower fraction of obese or overweight participants, lower rates of daily medication, allergies and less participants suffering from frequent bacterial infections prior to COVID-19. Scores of anxiety, depression and impairment of quality of life [3]⁠ were significantly higher in the IT than AT cohort (Table 1, Supplementary Fig. S3).

**Table 1:** Baseline characteristic of the Austria (AT) and Italy (IT) survey study cohorts

Variable^a^	AT	IT	Significance^b^	Effect size^b^
Sex	Female: 67% (n = 320) Male: 33% (n = 159)	Female: 70% (n = 300) Male: 30% (n = 127)	ns (p = 0.46)	V = 0.037
Education	Non-tertiary: 63% (n = 302) Tertiary: 37% (n = 176)	Non-tertiary: 59% (n = 250) Tertiary: 41% (n = 177)	ns (p = 0.35)	V = 0.047
Age, years	43 [IQR: 32–53] Range 18–80	45 [IQR: 34–54] Range 18–95	ns (p = 0.31)	r = 0.048
BMI before COVID-19	Normal: 54% (n = 257) Overweight: 28% (n = 135) Obesity: 18% (n = 84)	Normal: 66% (n = 278) Overweight: 25% (n = 104) Obesity: 8.8% (n = 37)	p = 0.0011	V = 0.15
Employment status	Employed: 83% (n = 398) Unemployed: 8.4% (n = 40) Leave: 1.7% (n = 8) Retired: 6.9% (n = 33)	Employed: 81% (n = 348) Unemployed: 9.4% (n = 40) Leave: 1.9% (n = 8) Retired: 7.3% (n = 31)	ns (p = 1)	V = 0.022
Autoimmunity	6.7% (n = 32)	6.3% (n = 27)	ns (p = 1)	V = 0.0072
Arterial hypertension	11% (n = 51)	8.4% (n = 36)	ns (p = 0.46)	V = 0.038
Pre-CoV depression/anxiety	5.4% (n = 26)	5.2% (n = 22)	ns (p = 1)	V = 0.0061
Diabetes	1.5% (n = 7)	0.23% (n = 1)	ns (p = 0.26)	V = 0.065
Freq. resp. infections	6.7% (n = 32)	3.3% (n = 14)	ns (p = 0.1)	V = 0.077
Cardiovascular disease	2.1% (n = 10)	3% (n = 13)	ns (p = 0.62)	V = 0.03
Hay fever/allergy	18% (n = 88)	12% (n = 51)	p = 0.045	V = 0.089
Malignancy	2.1% (n = 10)	4% (n = 17)	ns (p = 0.31)	V = 0.056
Gastrointestinal disease	1.7% (n = 8)	0.7% (n = 3)	ns (p = 0.46)	V = 0.044
Pulmonary disease	3.8% (n = 18)	2.8% (n = 12)	ns (p = 0.67)	V = 0.026
Freq. bact. infections	4.8% (n = 23)	1.2% (n = 5)	p = 0.016	V = 0.1
Pre-CoV sleep disorders	3.5% (n = 17)	4.7% (n = 20)	ns (p = 0.62)	V = 0.029
Daily medication	Absent: 62% (n = 295) 1–4 drugs: 37% (n = 175) 5 drugs and more: 1.9% (n = 9)	Absent: 74% (n = 317) 1–4 drugs: 25% (n = 106) 5 drugs and more: 0.94% (n = 4)	p = 0.0024	V = 0.14
Observation time	180 [IQR: 130–220] Range 90–400	140 [IQR: 120–270] Range 90–390	p = 0.0036	r = 0.12
Comorbidity	49% (n = 237)	43% (n = 185)	ns (p = 0.22)	V = 0.062

Numeric variables are presented as medians with interquartile ranges (IQR) and ranges. Categorical variables are presented as percentages and counts within the complete observation set

^a^BMI: body mass index, normal: BMI < 25 kg/m^2^, overweight: BMI 25–30 kg/m^2^, obesity: BMI > 30 kg/m^2^; Pre-CoV depression/anxiety: depression or anxiety before COVID-19; Freq. resp. infections: frequent (> 2 per year) respiratory infections;;Freq. bact. Infections: frequent (> two per year) bacterial infections with antibiotic therapy; Pre-CoV sleep disorders: sleep disorders before COVID-19

^b^Categorical variables: χ^2^ test with Cramer V effect size statistic. Numeric variables: Mann–Whitney U test with wilcoxon r effect size statistic. P values corrected form multiple testing with Benjamini–Hochberg method

CovILD study participants with the complete 1-year COVID-19 follow-up (n = 108, Supplementary Fig. S1) were predominantly male (59%). The median age was 56 (IQR: 49–68) years and 75% of the participants had comorbidities such as obesity, cardiovascular or pulmonary disease or type 2 diabetes mellitus. The CovILD study participants were stratified as mild (outpatients, 25%), moderate (inpatients, no ICU, 51%) and severe COVID-19 convalescents (ICU, 24%). The median age and comorbidity rates were significantly higher in moderate or severe than in mild COVID-19 patients (Table 2).

**Table 2:** Baseline characteristic of the CovILD study cohort and the study participants stratified by COVID-19 severity

Variable^a^	Entire cohort	Ambulatory CoV subset	Moderate CoV subset	Severe CoV subset	Significance^b^	Effect size^b^
Sex	Female: 41% (n = 44) Male: 59% (n = 64)	Female: 67% (n = 18) Male: 33% (n = 9)	Female: 35% (n = 19) Male: 65% (n = 36)	Female: 27% (n = 7) Male: 73% (n = 19)	p < 0.001	V = 0.31
Age, years	56 [IQR: 49–68] Range 19–87	47 [IQR: 38–55] Range 19–70	62 [IQR: 53–72] Range 27–87	56 [IQR: 52–64] Range 44–79	p < 0.001	η^2^ = 0.21
BMI at CoV onset	Normal: 39% (n = 42) Overweight: 43% (n = 46) Obesity: 19% (n = 20)	Normal: 56% (n = 15) Overweight: 33% (n = 9) Obesity: 11% (n = 3)	Normal: 29% (n = 16) Overweight: 51% (n = 28) Obesity: 20% (n = 11)	Normal: 42% (n = 11) Overweight: 35% (n = 9) Obesity: 23% (n = 6)	p < 0.001	V = 0.17
Comorbidity present	75% (n = 81)	41% (n = 11)	85% (n = 47)	88% (n = 23)	p < 0.001	V = 0.46
Cardiovascular disease	40% (n = 43)	7.4% (n = 2)	47% (n = 26)	58% (n = 15)	p < 0.001	V = 0.39
Arterial hypertension	27% (n = 29)	7.4% (n = 2)	27% (n = 15)	46% (n = 12)	p < 0.001	V = 0.31
Pulmonary disease	19% (n = 20)	11% (n = 3)	22% (n = 12)	19% (n = 5)	p = 0.031	V = 0.11
Metabolic disease	42% (n = 45)	19% (n = 5)	49% (n = 27)	50% (n = 13)	p < 0.001	V = 0.27
Diabetes II	15% (n = 16)	3.7% (n = 1)	15% (n = 8)	27% (n = 7)	p < 0.001	V = 0.23
Gastrointestinal disease	13% (n = 14)	0% (n = 0)	20% (n = 11)	12% (n = 3)	p < 0.001	V = 0.24
Malignancy	9.3% (n = 10)	3.7% (n = 1)	15% (n = 8)	3.8% (n = 1)	p < 0.001	V = 0.19
Immune deficiency	5.6% (n = 6)	0% (n = 0)	3.6% (n = 2)	15% (n = 4)	p < 0.001	V = 0.25

Numeric variables are presented as medians with interquartile ranges (IQR) and ranges. Categorical variables are presented as percentages and counts within the complete observation set

^a^BMI at CoV onset: body mass index at COVID-19 onset, normal: BMI < 25 kg/m^2^, overweight: BMI 25–30 kg/m^2^, obesity: BMI > 30 kg/m^2^

^b^Comparison of ambulatory, moderate and severe COVID-19 individuals. Categorical variables: χ^2^ test with Cramer V effect size statistic. Numeric variables: Kruskal–Wallis test with η^2^ effect size statistic. P values corrected form multiple testing with Benjamini–Hochberg method

### Longitudinal course of COVID-19 symptom resolution

**Fig. 1:**
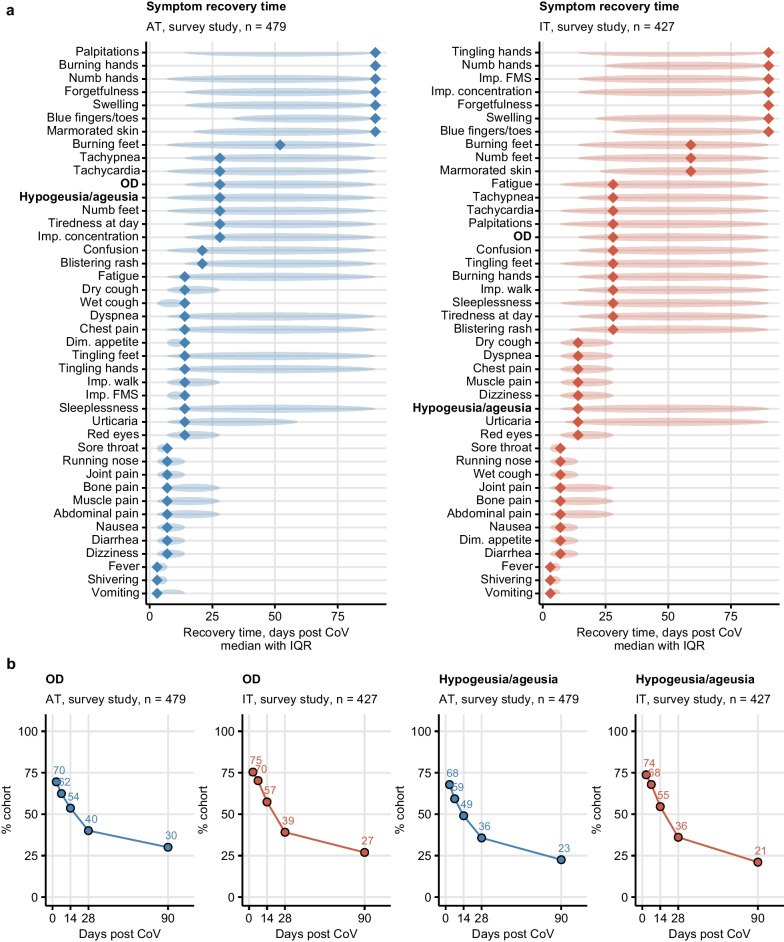
Symptom-specific recovery times in the ambulatory COVID-19 survey study. Symptom-specific recovery times were calculated for each participant of the survey study cohorts (Austria: AT, Italy: IT). **a** Distribution of the recovery times in the individuals with the indicated symptoms present during acute COVID-19 (first 14 days after clinical onset). Diamonds represent median recovery times, tinted ellipses code for interquartile ranges. Numbers of complete observations are indicated in the plot captions. **b** Percentages of individuals with self-reported olfactory dysfunction and taste disorders in the AT and IT survey study cohorts at particular time points after clinical onset. Numbers of complete observations are indicated under the plots. *OD* self-reported olfactory dysfunction, *Imp. concentration* impaired concentration, *Dim. appetite* diminished appetite, *Imp. walk* impaired walk, *Imp. FMS* impaired fine-motor skills

During acute infection of wild-type or alpha variant of SARS-CoV2, subjective OD (AT: 70%, IT: 75%) and self-reported taste disorders (AT: 68%, IT: 74%) along with fatigue, tiredness, diminished appetite, joint pain, tachypnea, cough, and fever were present in most survey participants (Supplementary Fig. S4). Fever, muscle and bone pain, shivering and eye redness, confusion, walk problems and tingling hands in the first 28 days of COVID-19 were significantly more frequent in the IT than AT cohort. In turn, dizziness was more common in the AT collective (Supplementary Fig. S5). Most upper airway and infection symptoms resolved within the first 14 days after clinical onset. Self-reported OD (median recovery: 28 days) and taste disorders (14 days) resolved substantially slower. Accordingly, self-reported OD (AT: 30%, IT: 27%) and taste disorders (AT: 22%, IT: 21%) affected more than one-fifth of convalescents 90 days after COVID-19 onset. Other symptoms with prolonged recovery included memory and concentration problems, tachypnea, tiredness and fatigue (Fig. 1, Supplementary Figs. S4 and S6, Supplementary Table S3).

Self-reported OD was frequent during acute COVID-19 in both inpatients and outpatients of the CovILD cohort (mild: 47%, moderate: 33%, severe COVID-19: 53%). The median recovery time across all disease severity strata was 90 days. Yet, subjective OD affected 16% of mild or moderate CovILD cohort individuals at the 360-day follow-up. By contrast, none of the severe COVID-19 patients reported subjective OD at the 180-day follow-up and beyond (Supplementary Figs. S7, S8, Supplementary Table S4).

### Subjective and objective OD during COVID-19 recovery

**Fig. 2:**
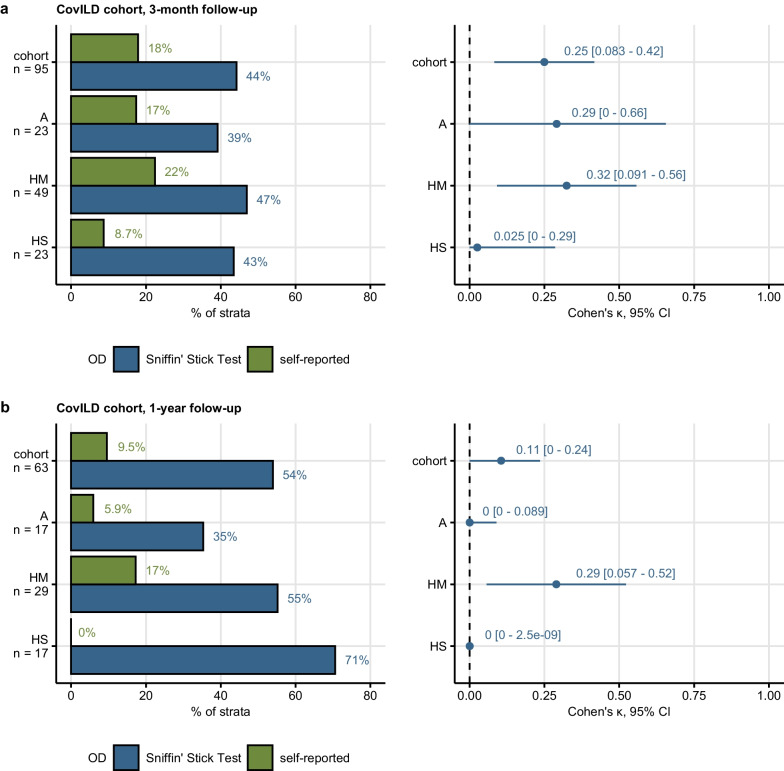
Rates of subjective and objective hyposmia in the CovILD cohort two months and one year after COVID-19. Objective olfactory dysfunction (OD) was diagnosed in CovILD study participants with < 13 correctly identified odorants in the 16-item Sniffin’ Sticks Identification Test. Frequencies of objective and self-reported olfactory dysfunction were compared at the 3-month (**a**) and 1-year follow-up (**b**) after COVID-19 in the entire cohort and the ambulatory (A), hospitalized moderate COVID-19 (HM) and hospitalized severe COVID-19 (HS) subset of the cohort. Concordance between the self-reported and objective olfactory dysfunction was assessed by Cohen’s $\upkappa$ inter-rater reliability statistic. Percentages of individuals with self-reported and objective hyposmia within the cohort or COVID-19 severity strata are presented in bar plots. Cohen’s $\upkappa$ with 95% confidence intervals (CI) are displayed in Forest plots

Objective OD was assessed in a subset of the CovILD cohort with the 16-item Sniffin’ Stick Test at the 3-month (n = 95) and 1-year follow-up (n = 63). Objective OD was diagnosed for < 13 odorants identified correctly [32–36]⁠. Objective OD in the entire analyzed CovILD cohort subsets (3-months: 45%, 1 year: 54%) and in their COVID-19 severity strata did not change over time. Furthermore, frequency of objective OD in the entire analyzed CovILD cohort subsets was higher than self-reported OD both at the 3-month (objective: 45%, subjective: 18%) and 1-year follow-up (objective: 54%, subjective: 9.5%). This was also true for each COVID-19 severity strata. Accordingly, the overall concordance between subjective and objective OD was low, as measured by Cohen’s κ (three months: κ = 0.25, 1 year: κ = 0.11). The highest concordance between objective and subjective OD was observed in moderate COVID-19 patients (three months: κ = 0.32, 1 year: κ = 0.29). Strikingly, objective OD affected 71% of severe COVID-19 survivors at the 1-year follow-up, while none of those individuals reported subjective OD (κ = 0) (Fig. 2). The discrepancy between self-reported and objective OD was corroborated by receiver-operating characteristic. For both investigated follow-ups, subjective OD was found to be an insensitive readout of objective OD, regardless of COVID-19 severity (three months: sensitivity 0.1–0.33, one year: sensitivity 0–0.31, Supplementary Figs. S9, S10).

Subsequently, we investigated changes in OD rating by Sniffin’ Stick Test and objective OD in CovILD study participants with complete testing results at both the 3-month and 1-year follow-up (n = 56, Supplementary Table S5). In an analysis of participant-matched testing results, we observed significant differences neither in numbers of correctly identified odorants nor in frequency of objective OD. Interestingly, 26.8% of all longitudinally investigated patients were diagnosed with objective OD at the 1-year follow-up despite normal olfactory function at the 3-month follow-up, suggestive of recurring or COVID-19-independent OD (Supplementary Fig. S11).

### Subjective OD and taste disorders as distinct post-acute sequelae of COVID-19

**Fig. 3:**
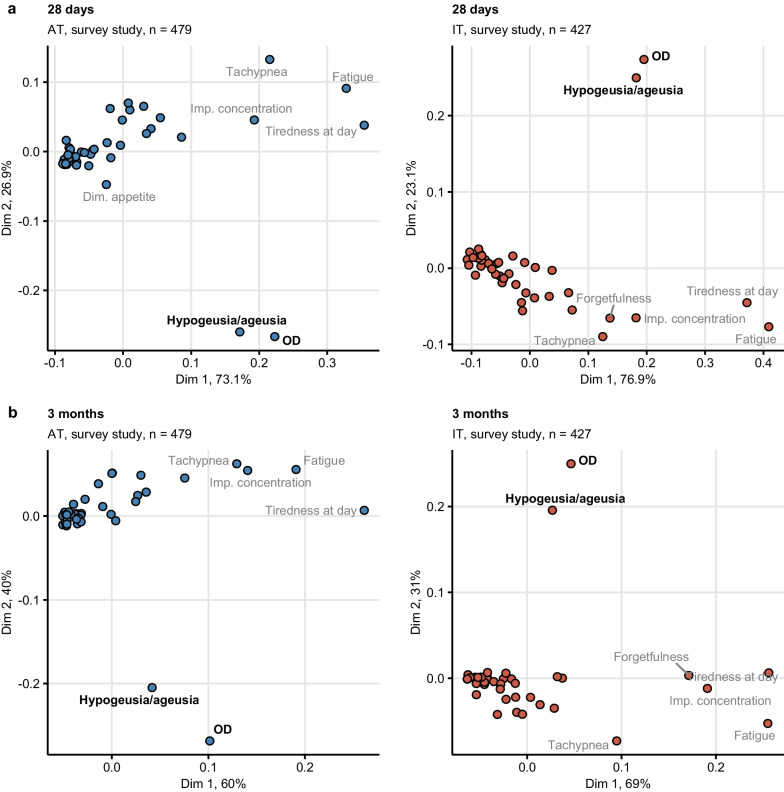
Self-reported olfactory dysfunction and taste disorders are isolated persistent symptoms of COVID-19. Symptom data during at 28 days (**a**) and 3 months (**b**) after clinical onset in the Austria (AT) and Italy (IT) survey study cohorts were subjected to two-dimensional multi-dimensional scaling (MDS) with simple matching distance between the symptoms. MDS coordinates are presented in scatter plots. Selected data points are labeled with the symptom names. Percentages of the data set variance associated with the MDS dimensions are indicated in the plot axes. Numbers of complete observations are indicated in the plot captions. *OD* self-reported olfactory dysfunction, *Imp. concentration* impaired concentration

During acute COVID-19 in the survey study, self-reported OD was frequently accompanied by taste disorders and multiple non-specific infection symptoms such as diminished appetite, rhinitis and sore throat, as found by multi-dimensional scaling (Supplementary Fig. S12). During recovery, this association disappeared owing to the resolution of most acute infection symptoms. At 28 days and three months after clinical onset, subjective OD and taste disorders were mapped close to each other and far from other leading post-acute symptoms including tachypnea, fatigue, memory and concentration deficits (Fig. 3). This indicates that, post-acute subjective OD and taste disorders are rarely accompanied by other persistent symptoms.

The co-occurrence of OD and taste disorders was supported by association rule mining [40]⁠ in the survey study, where ≥ 82% and ≥ 66% of participants reporting OD at 28 days and three months after clinical onset, respectively, were affected by taste disorders as well. Furthermore, the combination of subjective OD and taste disorders were found in one-third of survey participants at 28 days and one-fifth of participants at three months after COVID-19 onset (Supplementary Fig. S13).

### Smell and taste disorder phenotype of COVID-19 recovery

Three subsets of COVID-19 convalescents were identified by PAM clustering [38]⁠ of the training AT survey cohort by resolution times of particular symptoms. As assessed by permutation analysis, tiredness, fatigue, concentration and memory problems, tachypnea, self-reported OD and taste disorders were the most important symptoms for the cluster definition (Supplementary Fig. S15). Subsequently, the IT test cohort individuals were assigned to the clusters with a nearest-neighbor classification procedure [39]⁠. The clustering scheme was highly reproducible as inferred from comparable fractions of explained clustering variances (AT: 0.59, IT: 0.56) and similar distribution of the cluster sizes in the training AT and test IT cohort (Supplementary Fig. S16).

**Fig. 4:**
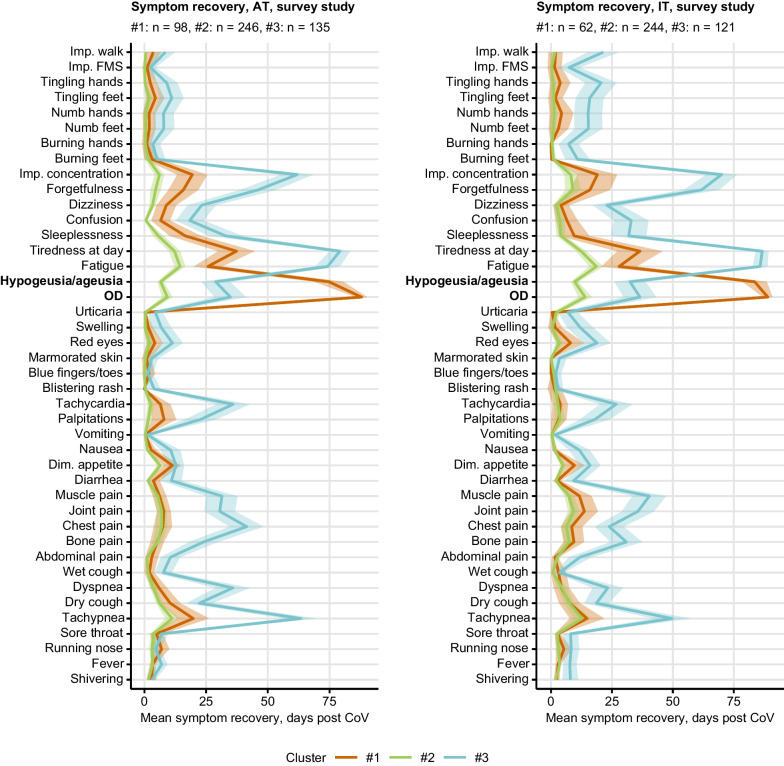
Differing duration of neurocognitive and respiratory symptoms, fatigue, olfactory dysfunction and taste disorders defines the COVID-19 recovery clusters. Clustering of the survey study participants in respect to symptom-specific recovery times was done by semi-supervised PAM algorithm (partitioning around medoids, Euclidean distance, training cohort: Austria [AT], test cohort: Italy [IT]). Mean recovery times in the recovery clusters are presented as lines, 2 $\times$ SEM intervals are displayed as tinted regions. Numbers of individuals assigned to the recovery clusters are indicated in the plot captions. *OD* self-reported olfactory dysfunction, *Dim. appetite* diminished appetite, *Imp. concentration* impaired concentration, *Imp. walk* impaired walk, *Imp. FMS* impaired fine motor skills

The hallmark of cluster #1 (AT: 21%, IT: 15% of participants) was the slowest resolution of self-reported OD and taste disorders. Most other symptoms including tiredness, fatigue, respiratory, memory and concentration difficulties resolved rapidly in cluster #1. The largest cluster #2 comprised > 50% of participants (AT: 51%, IT: 56%) and was characterized by both the fastest resolution of all surveyed symptoms and the lowest number of acute COVID-19 symptoms. By contrast, cluster #3 (AT: 28%, IT: 29%) displayed the slowest resolution of fatigue, tiredness, tachypnea, memory and concentration deficits and the highest number of acute and post-acute COVID-19 complaints (Fig. 4, Supplementary Fig. S16).

Concerning demographic and clinical background, cluster #3 included the oldest participants with the highest comorbidity and daily medication rates. Clusters #1 and #2 consisted of substantially younger participants with similarly low frequencies of comorbidities. Females were significantly over-represented in clusters #1 (AT: 79%, IT: 84%) and #3 (AT: 76%, IT: 77%) (Supplementary Fig. S17, Supplementary Tables S6, S7).

**Fig. 5:**
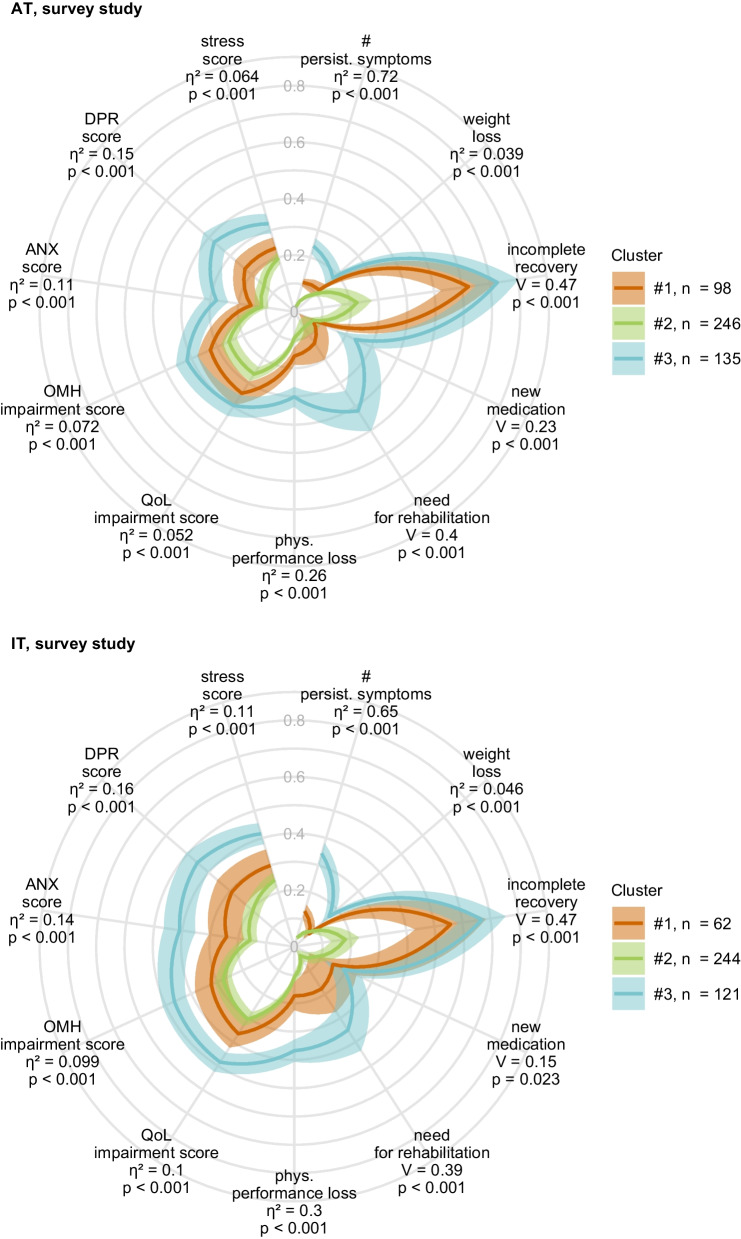
Physical and mental health, and quality of life in the COVID-19 recovery clusters. Clustering of the survey study participants in respect to symptom-specific recovery times was done by the semi-supervised PAM algorithm (partitioning around medoids, Euclidean distance, training cohort: Austria [AT], test cohort: Italy [IT]). Minimum/maximum scaled readouts of clinical and physical recovery, mental health and quality of life at the time of survey completion in the clusters in the Austria (AT) and Italy (IT) survey study cohorts are presented. Dichotomous items (incomplete convalescence, weight loss, new medication and need for rehabilitation) were binarized (yes: 1, no: 0) prior to visualization. Statistical significance for differences between the clusters was assessed by Kruskal–Wallis with ${\upeta }^{2}$ effect size statistic (numeric variables) or ${\upchi }^{2}$ test with Cramer V effect size statistic (categorical variables). P values were corrected for multiple testing with Benjamini–Hochberg method. Lines represent mean values, 2 $\times$ SEM intervals are displayed as tinted regions. Effect sizes and p values are shown in the plots. Numbers of individuals assigned to the recovery clusters are indicated in the plot legends. Incomplete recovery: self-reported incomplete recovery; # persist. symptoms: number of symptoms at 28 days after clinical onset; phys. Performance loss: physical performance loss as compared with the time before COVID-19; QoL impairment score: score of impaired quality of life; OMH impairment score: overall mental health impairment score; *ANX score* anxiety score, Patient Health Questionnaire, PHQ-4; *DPR* depression score, Patient Health Questionnaire, PHQ-4; stress score: mental stress score, 7 item PHQ stress module

Weight and physical performance loss as well as rates of new medication and need for rehabilitation were highest in cluster #3. Furthermore, cluster #3 individuals had the worst scoring of anxiety, depression, mental stress, self-reported mental health and quality of life at the time of survey completion. In comparison, those psychometric readouts were significantly better in cluster #1 despite slow recovery from OD and taste deficits and in cluster #2 with the fastest symptom resolution (Fig. 5, Supplementary Tables S8, S9).

## Discussion

Herein, we demonstrated that subjective OD is a frequent acute and post-acute symptom in non-hospitalized (47–75% patients) and hospitalized COVID-19 patients (33–53%) that were infected with the wild-type or alpha SARS-CoV-2 variant, as reported previously [4, 6, 9, 10, 20]. As evident from our Sniffin’ Stick Test results, subjective OD may systematically underestimate the frequency of objective OD in COVID-19 convalescents. During recovery from ambulatory COVID-19, self-perceived persistent OD was accompanied by taste disorders but not by other common post-acute sequelae such as fatigue, tiredness, tachypnea, memory and concentration deficits. Patients with a slow resolution of OD and taste disorders but good recovery from other symptoms comprised predominantly young females with low comorbidity rates, good mental health, and high rating of physical performance. These findings may qualify persistent and isolated smell and taste disorders as a distinct phenotype of COVID-19 recovery.

In the survey and the observational CovILD cohort, percentages of self-perceived OD halved during the first four weeks of convalescence. Still, every sixth mild or moderate COVID-19 patient in the observational setting reported OD at the 1-year follow-up. This resembles the previously reported bi-phasic kinetic with fast OD resolution within the first few weeks followed by a plateau [14, 16, 19, 21, 27, 41]⁠. Self-reported OD was described in 10% of convalescents 2 years after COVID-19 [15]⁠. Objective OD, as measured by the Sniffin’ Stick Test, was found in 3% of convalescents at the 1-year follow-up [27, 32]. This indicates, that the complete recovery from post-COVID-19 OD may take months to years, comparable to other viral diseases or traumatic brain injury (TBI) [42, 43]⁠. Similar to other viral infections and TBI [42]⁠, olfactory training may accelerate recovery from post-COVID-19 OD [25–27].

Smell function testing results in the CovILD cohort suggest that self-reported OD is not a sensitive readout of objective OD and underestimates the true frequency of OD. This phenomenon may be partially attributed to objective, COVID-19-independent OD, whose prevalence is estimated to be as high as 29% in the general population and to rise with age [35, 44]⁠. Discordant objective and subjective OD has been described in the normal population [44]⁠ and even individuals with functional anosmia report normal olfactory function [45]⁠. In our analysis, the discrepancy between objective and subjective OD was the most striking in severe COVID-19 survivors during long-term recovery and may be explained by co-occurring critical illness neuropathy in these patients. Furthermore, results of our longitudinal OD testing suggest de-novo development or recurrence of objective OD in a subset of COVID-19 patients. Of note, a recurrent pattern of OD during COVID-19 recovery was recently reported [16]⁠. Collectively, these findings advocate standardized functional screening tools and longitudinal study design to accurately assess OD during COVID-19 convalescence.

Heterogeneity of post-COVID-19 condition manifests by phenotypes with distinct symptom patterns [1, 6]⁠. Characterization of such phenotypes is crucial for prediction of individual outcomes and rehabilitation needs. We found that subjective post-acute OD co-occurred with self-reported taste disorders in > 66% of convalescents, which is likely a result of an impaired retronasal smell [19]⁠. Importantly, those two symptoms were rarely accompanied by other persistent complaints, especially three months after COVID-19 onset. This persistent smell and taste disorder phenotype observed in cluster #1 participants of the survey study may be regarded as a distinct form of COVID-19 sequelae affecting preferentially younger, female individuals without pre-existing chronic conditions. Previous studies described particularly high rates of acute OD [19]⁠ and general significantly slower recovery from OD in females than males [14]⁠. Consequently, female patients with OD during acute COVID-19 may best benefit from early neurological and/or laryngology assessment and timely initiated therapy.

Multiple reports linked post-COVID-19 OD to impaired quality of life, anxiety, and depression [20, 23, 24, 33]⁠. In our study, cluster #1 smell and taste disorder phenotype was characterized by higher ratings of quality of life, physical and mental health. This may be best explained by the fact that individuals with post-acute OD had low frequencies of persistent fatigue, neurocognitive and respiratory sequelae which interfere severely with daily functioning and professional activity. Along this line, the ‘slow recovery phenotype’ in cluster #3 with fatigue, memory and concentration problems displayed the worst performance status, mental health, and quality of life. Still, the discrepant effects of OD on quality of life may be in part attributed to methodological differences as our assessment battery did not address OD-related quality of life measures.

Independently recruited collectives of COVID-19 convalescents and application of contemporary machine learning algorithms are the main strengths of our analysis approach. In particular the survey study included two independently recruited cohorts in two countries with different containment measures and dynamics of SARS-CoV-2 outbreaks. Infection timepoint, follow-up time, readouts of mental health and quality of life were the key features discriminating between the AT and IT cohorts [1, 3]⁠. Still, we could validate the COVID-19 recovery clusters defined in the AT collective and corroborate the effects of cluster assignment on physical and mental health in the IT cohort.

Our study bears limitations. Females and health care workers were over-represented in the survey study indicating a selection bias towards health-aware individuals with post COVID condition. Short follow-up and retrospective record of symptoms as pre-defined classes in the survey study likely limited the analysis precision and precluded investigation of symptom relapses [1, 3]⁠. In the CovILD cohort a possible dropout of participants with subjective complete recovery was likely a source of selection bias [28]⁠. Next, we could not investigate effects of immunization, improved medication and the most recent SARS-CoV-2 variants in our study cohorts recruited at the initial phase of the pandemic during the wild-type and alpha variant outbreaks. In particular, OD following an omicron-variant SARS-CoV-2 infection is reportedly less frequent as compared with the wild type, alpha or delta pathogen [8, 9]⁠. Hence, there is a continuous need for phenotyping of COVID-19-related OD as new variants of concern emerge. In addition, the 16-item Sniffin’ Sticks Identification test we used is not an international standard, while the UPSIT has been validated in huge collectives. This limits the comparability of our study. Finally, we found a high discrepancy between subjective and objective OD, especially after severe COVID-19 infection. Objective OD data were available only for the CovILD collective. Hence, self-reported OD may underestimate the true OD rate in the survey study and limit its interpretation.

## Conclusion

Our multi-cohort analysis describes slow-pace resolution of subjective OD both in COVID-19 inpatients and outpatients. Except for taste disorders, persistent OD was rarely accompanied by other post-acute sequelae such as fatigue, tachypnea, or neurocognitive manifestations. In contrast to the COVID-19 convalescents with a slow recovery from fatigue and neurocognitive complaints, the subset affected by isolated OD and taste disorders was characterized by the absence of physical or mental health deficits. This suggests that smell and taste disorder phenotype of post-acute COVID-19 sequelae may pose a minor challenge for public health. Finally, our results stress the heterogeneity of post-COVID-19 condition requiring tailored management strategies.

### Supplementary Information

Below is the link to the electronic supplementary material.

Supplementary file1 (PDF 11211 KB)

Supplementary file2 (XLSX 19 KB)

## Data Availability

The data that support the findings of this study are available from the corresponding authors upon reasonable request. The entire analysis pipeline was published at https://github.com/PiotrTymoszuk/hyposmia_analysis_pipeline.
