# Supplementary material for: Distinct smell and taste disorder phenotype of post-acute COVID-19 sequelae
Source: Eur Arch Otorhinolaryngol. 2023 Sep 5;280(11):5115–28. doi: 10.1007/s00405-023-08163-x (PMC10562286; doi:10.1007/s00405-023-08163-x)
Supplement: Supplementary file 1 — Supplementary file1 (PDF 11211 KB) [file 405_2023_8163_MOESM1_ESM.pdf]

Distinct smell and taste disorder phenotype of post-acute COVID-19 sequelae

Supplementary Material

Health after COVID-19 in Tyrol and CovILD study teams

2023-05-30

Supplementary Methods

Study procedures and variables

The complete list of study variables and stratification scheme is provided in **Supplementary Table S1** for the Health after COVID-19 in Tyrol survey study and in **Supplementary Table S2** for the observatory CovILD cohort.

COVID-19 symptoms

A total of 42 self-reported symptoms were recorded in the survey study cohorts (**Supplementary Figure S4** and **Supplementary Table S1**). The symptom duration was coded as follows: absent: 0 days, 1 - 3 days: 3 days, up to 1 week: 7 days, up to 2 weeks: 14 days, up to 4 weeks: 28 days, up to or greater than 3 months: 3 months. Acute symptoms were defined as complaints present during the first 14 days after clinical onset of COVID-19.

In the observatory CovILD cohort, a total of 8 self-reported symptoms (reduced physical performance, olfactory dysfunction, dyspnea, sleep problems, cough, fever, night sweating, gastrointestinal symptoms) were recorded with a standardized questionnaire at each of 60-, 100-, 180- and 360-day post COVID-19 follow-up (**Supplementary Figure S7** and **Supplementary Table S2**). Acute COVID-19 symptoms were assessed retrospectively at the 60-day follow-up [1, 2].

Rating of physical recovery, mental health and quality of life in the survey study

Self-perceived complete recovery, rehabilitation need and new medication since COVID-19 at the time of study participation were surveyed as single yes/no items. Percentage of physical performance loss as compared with the time before COVID-19 was rated with a 0 - 100% scale [3, 4]. Quality of life impairment (QoL) and overall mental health impairment (OMH) were rated with a 4 item Likert scale each (possible answers: “excellent,” “good,” “fair,” “poor,” scored: 0, 1, 2, and 3) [3, 4]. Anxiety (ANX) and depression (DPR) were assessed with the Patient Health Questionnaire (PHQ-4) [3–5]. Mental stress was scored with a modified 7 item PHQ stress module as described before [3, 4, 6].

Rating of olfactory dysfunction with Sniffin’ Stick Test

Objective olfactory dysfunction at the 100-day and 360-day follow-up in the CovILD study participants was investigated with the 16-item Sniffn’ Stick Identification Test as described [7–10]. In brief, the nasal chemosensory performance was investigated using pen-like odor-dispensing devices for odor identification of 16 common odorants (multiple forced-choice from four verbal items per test odorant). Clinically relevant olfactory dysfunction was defined as < 13 correct answers (points) [7, 11]. In the analysis, participants with the

complete answers concerning self-reported olfactory dysfunction and complete test results were included.

Data analysis

Software

Analysis was done with R version 4.2.3 (The R Project for Statistical Computing).

Import of raw study data was accomplished with the packages *foreign* [12] and *readxl* [13]. Tabular data was handled with the *tidyverse* package bundle [14], and the *rlang* [15] and *trafo* packages. Search and transformation of text data was done with *stringi* [16]. For exploratory data analysis, descriptive statistic and statistical hypothesis testing, packages *rstatix* [17], *vcd* [18], *rcompanion* [19] and *ExDA* were used. For modeling of symptom recovery, packages *lme* [20], *lmerTest* [21] and *kinet* were employed. Multi-dimensional scaling was performed with the base R's *stats* package and *clustTools*. Apriori analysis was done with *arules* [22]. Clustering analysis was performed with the packages *cluster* [23], *philentropy* [24], *scrime* [25], *factoextra* [26] and *clustTools*. Inter-rater analysis and receiver operating characteristic (ROC) were done with the packages *caret* [27] and *vcd* [18]. Parallelization of analysis tasks was accomplished with the package *furrr* package [28].

Analysis results were visualized with *ggplot* [29] (bar, scatter and bubble plots, heat maps of confusion matrices), *ExDA* (ellipse plots, radar, ribbon, stack and violin plots), *kinet* (kinetic of symptom resolution), *plotROC* [30] (ROC plots), *clustTools* (multi-dimensional scaling projection/scatter plots, heat maps of clustering features, within-cluster sum of squares). HTML elements within the plots were handled with tools provided by the package *ggtext* [31].

Figures were generated with *cowplot* [32]. Tables were rendered with the *flextable* package [33]. Supplementary Material and parts of the manuscript were written in the *rmarkdown* environment [34] and were rendered as Word documents with the *knitr* [35] and *bookdown* [36] packages. Management of figures and tables within the *rmarkdown* documents was accomplished with the development package *figur*.

Data import and formatting

Data of the survey study [3, 4] were imported from SPSS files with the *foreign* package (function `read.spss()`). The CovILD study data set [37] was imported from an Excel file with the function `read_xlsx()` (package *readxl*). Formatting of the raw data sets was done with in-house developed scripts available from the project's GitHub repository.

Estimation of minimal sample size for clustering analysis

To assess the minimal sample size of the survey study required for reliable clustering analysis, random subsets of the pooled Austria and Italy cohort COVID-19 symptom data

set with differing observation numbers (50, 100, 200, 300, 400, 500, 600, 700, 800, 900 observations, 20 random subsets per sample size) were generated and their clustering tendency was assessed with Hopkins statistic (H). Possible H values span from 0 to 1, where H = 0 indicates an ideal uniform distribution and H = 1 suggests a highly clustered data. Beginning from the sample size of n = 400, no improvement of the clustering tendency (plateau) could be observed. This suggest n = 400 as minimal sample size required for reproducible clustering analysis results (**Supplementary Figure S2**). Given the size of the Austria and Italy cohorts (n = 479 and n = 427, respectively) and their H values being very close to the H plateau value in the random subset analysis (Austria: H = 0.8, Italy: H = 0.79), we inferred that the size of single study cohorts was sufficient for reliable and reproducible clustering analysis and hence abstained from pooling the survey study cohorts.

Descriptive statistic and effect size

If not indicated otherwise, numeric values are presented in the manuscript text and tables as medians with interquartile ranges (IQR) and ranges. Qualitative variables are presented as percentages and counts of their categories within the complete observation set. Descriptive statistic values were computed with the function `explore()` from the *ExDA* package.

Effects sizes were assessed following the scheme proposed by Cohen [38]. Effect size for comparison of categorical variables was measured with Cramer's V statistic (weak: < 0.3, moderate: 0.3 - 0.5, large effect: ≥ 0.5). Effect size for two-group comparisons of numeric variables was assessed with r statistic (weak: < 0.3, moderate: 0.3 - 0.5, large effect: ≥ 0.5). Effect size for multi-group comparison of numeric variables was assessed with η^2 (weak: < 0.13, moderate: 0.13 - 0.26, large effect: ≥ 0.26). Effect size for comparison of paired proportions (2 × 2 contingency table) was assessed with Cohen's g (weak: < 0.15, moderate: 0.15 - 0.25, large effect: ≥ 0.25). Effect size for comparison of study participant-matched proportions (multi-dimensional contingency table) was assessed with Kendall's W (weak: < 0.3, moderate: 0.3 - 0.5, large effect: ≥ 0.5). Effect size for inter-rate reliability was measures with Cohen's κ statistic was interpreted as follows: 0.01 – 0.20 as none to slight, 0.21 – 0.40 as fair, 0.41 – 0.60 as moderate, 0.61 – 0.80 as substantial, and 0.81 – 1.00 as strong [39]. Effect sizes were computed with the function `compare_variables()` from the development package *ExDA* employing tools introduced by the *rstatix* and *vcd* packages (Cramer's V, r, η^2, Cohen's κ, Cohen's g) or with the function `cohenG()` from the package *rcompanion* [19].

Statistical hypothesis testing and inter-rater reliability

Since multiple study variables were non-normally distributed as assessed by Shapiro-Wilk test and visual assessment of their distribution (quantile - quantile plots), statistical significance for differences in outcome numeric variables were assessed with Mann-Whitney U test with r effect size statistic (two groups, independent data), paired Wilcoxon test with r effect size statistic (two groups, paired data) or Kruskal-Wallis test with η^2 effect

size statistic. Differences in frequency distribution for categorical outcome variables were assessed by χ^2 test with Cramer V effect size statistic. Significance of differences of paired proportions (2×2 contingency table) was determined with McNemar test with Cohen's g effect size statistic. Mann-Whitney, Kruskal-Wallis, χ^2 tests were accomplished with the function `compare_variables()` (package *ExDA*) employing algorithms from the package *rstatix* [17]. McNemar tests and Cohen's g were calculated with the functions `mcnemar_test()` (package *rstatix*) [17] and `cohenG()` (package *rcompanion*) [19], respectively. Significance of symptom resolution (longitudinal, participant-matched binary data) was determined by Cochran's Q test with Kendall's W effect size statistic (function `cochran_qtest()`, package *rstatix*) [17, 40].

Inter-rater assessment of self-reported and Sniffin Test olfactory dysfunction was accomplished with Cohen's κ statistic [39, 41]. κ significance ($\kappa \neq 0$) was estimated with Wald Z test (function `correlate_variable()`, package *ExDA* employing the *vcd* package) [18]. Additionally, reliability of detection of objective olfactory dysfunction (Sniffin' Stick Test) was additionally assessed by ROC analysis. Overall accuracy, sensitivity and specificity was computed with the functions `defaultSummary()` and `twoClassSummary()` from the package *caret* [27].

P values were adjusted for multiple testing with Benjamini-Hochberg method [42] separately for each analysis task and cohort. Effects with $p < 0.05$ were considered significant.

Symptom-symptom distances and multi-dimensional scaling

To assess co-occurrence or exclusivity of symptoms, simple matching distances between manifestations during the first 14 days, at 28 days and at 3 months after clinical onset in the survey study cohorts were calculated (function `calculate_dist()`, package *clustTools* employing tools from packages *scrime* and *philentropy*) [24, 25, 43]. Subsequently, the distance matrix was subjected to multi-dimensional scaling (MDS, k = 2 dimensions, package *stats*, function `cmdscale()`). Association of specific symptoms was assessed by visual analysis of MDS coordinate plots.

Apriori analysis of COVID-19 symptoms in the survey study

Frequent combinations of symptoms during the first 14 days, at 28 days and at 3 months after clinical onset in the survey study cohorts were identified with the apriori algorithm (function `apriori()`, package *arules*) [22, 44] with the minimal support cutoff of 0.15, 2 - 10 item transaction length, confidence > 0.6 and lift > 2. The support statistic were used to estimate the symptom combination frequency. The confidence value was treated as an estimate of conditional probability of the symptom co-occurrence. The lift statistic was interpreted as a measure of the symptom dependence (lift = 1, symptoms are independent). Frequency of symptom combination in the survey study cohorts and

percentage of co-occurrence of the symptoms within the given symptom combination were displayed in bobble plots.

Clustering analysis

COVID-19 recovery clusters of the training Austria survey cohort participants in respect to symptom-specific recovery times (**Figure 1A**) were defined with the PAM (partitioning around medoids) algorithm and Euclidean distance statistic [23, 24]. The set of participants with the complete clustering variable set (COVID-19 symptom recovery times) was included in the analysis. The symptom recovery times were not subjected to any type of pre-processing. The clustering objects were generated with the function `kcluster(clust_fun = 'pam')` from the package *clustTools*.

The choice of the clustering procedure was motivated by the analysis of the fraction of explained clustering variance (ratio of the total between-cluster to total sum of squares) and clustering structure stability in 10-fold cross-validation (metric: rate of correct cluster assignment, cluster assignment predicted by inverse distance-weighted 7-nearest neighbors label propagation algorithm) [45, 46] for several clustering algorithms as presented in **Supplementary Figure S14A**. The fractions of explained clustering variance and cross-validated cluster assignment accuracy were calculated with the methods `var()` and `cv()` from the package *clustTools*.

The optimal number of clusters was determined by the bend of the total within-cluster sum of squares curve (**Supplementary Figure S14B**, method `plot()`, package *clustTools*, employing the genuine *factoextra* algorithm) [26]. Permutation importance of specific clustering variables was investigated by calculating difference in clustering variance (ratio of total between-cluster sum of squares to total sum of squares) between the initial clustering object and the clustering object with the given variable reshuffled at random (function `impact()`, package *clustTools*). The permutation importance statistics were computed for 20 random permutations of each clustering variable.

Assignment of the Italy survey cohort participants to the recovery clusters was accomplished with the inverse distance-weighted 7-nearest neighbors label propagation classifier [46]. The clustering efficacy in the training Austria cohort and the test Italy cohort measured by clustering variance statistic defined above was similar (Austria: 0.59, Italy: 0.56), which indicate good reproducibility of the clustering structure developed in the training Austria cohort.

Data and source code availability

The raw data files will be made available upon request. The entire analysis pipeline was published at https://github.com/PiotrTymoszuk/hyposmia_analysis_pipeline.

Supplementary Tables

Supplementary Table S1: Survey study variables. The table is available as a supplementary Excel sheet.

Supplementary Table S2: CovILD study variables. The table is available as a supplementary Excel sheet.

Supplementary Table S3: Results of statistical hypothesis testing for significant recovery of the most frequent COVID-19 symptoms in the Austria (AT) and Italy cohort (IT) of the survey study.

**Cohort**	**Symptom**[a]	**Significance**[b]	**Effect size**[b]
AT	Fatigue	$p < 0.001$	W = 0.43
	Tiredness at day	$p < 0.001$	W = 0.33
	OD	$p < 0.001$	W = 0.26
	Hypogeusia/ageusia	$p < 0.001$	W = 0.29
	Joint pain	$p < 0.001$	W = 0.37
	Dim. appetite	$p < 0.001$	W = 0.41
	Tachypnea	$p < 0.001$	W = 0.22
	Muscle pain	$p < 0.001$	W = 0.32
	Fever	$p < 0.001$	W = 0.38
IT	Fatigue	$p < 0.001$	W = 0.36
	Tiredness at day	$p < 0.001$	W = 0.3
	OD	$p < 0.001$	W = 0.33
	Hypogeusia/ageusia	$p < 0.001$	W = 0.35
	Joint pain	$p < 0.001$	W = 0.39
	Dim. appetite	$p < 0.001$	W = 0.38
	Tachypnea	$p < 0.001$	W = 0.2
	Muscle pain	$p < 0.001$	W = 0.36
	Fever	$p < 0.001$	W = 0.52

[a]OD: self-reported olfactory dysfunction; Dim. appetite: diminished appetite.

[b]Cochran's Q test with Kendall's W effect size statistic. P values corrected for multiple testing with Benjamini-Hochberg method.

Supplementary Table S4: Results of statistical hypothesis testing for significant recovery of COVID-19 symptoms in COVID-19 severity strata of the CovILD study.

**COVID-19 severity**	**Symptom**[a]	**Significance**[b]	**Effect size**[b]
ambulatory	Sleep problems	$p = 0.048$	$W = 0.11$
	Dyspnea	$p < 0.001$	$W = 0.23$
	Cough	$p < 0.001$	$W = 0.28$
	Fever	$p < 0.001$	$W = 0.42$
	Night sweat	$p = 0.0095$	$W = 0.14$
	Gastrointestinal	$p < 0.001$	$W = 0.27$
	OD	$p = 0.0044$	$W = 0.19$
	Reduced performance	$p < 0.001$	$W = 0.38$
moderate	Sleep problems	ns ($p = 0.87$)	$W = 0.0069$
	Dyspnea	$p < 0.001$	$W = 0.17$
	Cough	$p < 0.001$	$W = 0.44$
	Fever	$p < 0.001$	$W = 0.76$
	Night sweat	$p < 0.001$	$W = 0.27$
	Gastrointestinal	$p < 0.001$	$W = 0.23$
	OD	$p = 0.032$	$W = 0.06$
	Reduced performance	$p < 0.001$	$W = 0.27$
severe	Sleep problems	ns ($p = 0.89$)	$W = 0.018$
	Dyspnea	$p < 0.001$	$W = 0.37$
	Cough	$p < 0.001$	$W = 0.38$
	Fever	$p < 0.001$	$W = 1$
	Night sweat	$p < 0.001$	$W = 0.31$
	Gastrointestinal	ns ($p = 0.14$)	$W = 0.1$
	OD	$p < 0.001$	$W = 0.38$

COVID-19 severity	Symptom[a]	Significance[b]	Effect size[b]
	Reduced performance	p = 0.0023	W = 0.27

[a]OD: self-reported olfactory dysfunction.

[b]Cochran's Q test with Kendall's W effect size statistic. P values corrected for multiple testing with Benjamini-Hochberg method.

Supplementary Table S5: Results of the Sniffin' Stick Test in the CovILD study subset with the complete longitudinal follow-up data. Numeric variables are presented as medians with interquartile ranges (IQR) and ranges. Categorical variables are presented as percentages and counts within the complete observation set.

**Variable**[a]	**3-month follow-up**	**1-year follow-up**[b]	**Significance**[b]	**Effect size**
Participants, n	56	56		
Sniffin' Stick Test, points	13 [IQR: 12 - 14]<br>range: 0 - 16	12 [IQR: 11 - 14]<br>range: 3 - 16	ns (p = 0.16)	r = 0.2
Sniffin' Stick Test OD, < 13 points	38% (n = 21)	50% (n = 28)	ns (p = 0.42)	g = 0.15

[a]OD: olfactory dysfunction.

[b]Categorical variables: McNemar test with Cohen's g effect size statistic. Numeric variables: paired Wilcoxon test with r effect size statistic. P values corrected for multiple testing with Benjamini-Hochberg method.

Supplementary Table S6: Demographic and baseline clinical characteristic at the COVID-19 onset of the survey study participants assigned to the recovery clusters, Austria (AT) cohort. Numeric variables are presented as medians with interquartile ranges (IQR) and ranges. Categorical variables are presented as percentages and counts within the complete observation set.

**Variable**[a]	**Cluster #1**	**Cluster #2**	**Cluster #3**	**Significance**[b]	**Effect size**[b]
Sex	female: 79% (n = 77)<br>male: 21% (n = 21)	female: 57% (n = 140)<br>male: 43% (n = 106)	female: 76% (n = 103)<br>male: 24% (n = 32)	p < 0.001	V = 0.22
Age, years	42 [IQR: 30 - 50]<br>range: 21 - 80	43 [IQR: 29 - 53]<br>range: 18 - 77	48 [IQR: 38 - 53]<br>range: 21 - 70	p = 0.045	$\eta^2 = 0.012$
BMI before COVID-19	normal: 62% (n = 60)<br>overweight: 24% (n = 23)<br>obesity: 14% (n = 14)	normal: 55% (n = 133)<br>overweight: 29% (n = 70)<br>obesity: 17% (n = 41)	normal: 47% (n = 64)<br>overweight: 31% (n = 42)<br>obesity: 21% (n = 29)	ns (p = 0.39)	V = 0.073
Education	non-tertiary: 64% (n = 62)<br>tertiary: 36% (n = 35)	non-tertiary: 63% (n = 154)<br>tertiary: 37% (n = 92)	non-tertiary: 64% (n = 86)<br>tertiary: 36% (n = 49)	ns (p = 0.99)	V = 0.012
Employment status	employed: 87% (n = 85)<br>unemployed: 7.1% (n = 7)<br>leave: 3.1% (n = 3)<br>retired: 3.1% (n = 3)	employed: 80% (n = 198)<br>unemployed: 9.3% (n = 23)<br>leave: 1.6% (n = 4)<br>retired: 8.5% (n = 21)	employed: 85% (n = 115)<br>unemployed: 7.4% (n = 10)<br>leave: 0.74% (n = 1)<br>retired: 6.7% (n = 9)	ns (p = 0.56)	V = 0.079
Observation time	180 [IQR: 130 - 210]<br>range: 93 - 400	190 [IQR: 130 - 220]<br>range: 90 - 400	180 [IQR: 140 - 220]<br>range: 90 - 380	ns (p = 0.85)	$\eta^2 = -0.0029$
Comorbidity	46% (n = 45)	44% (n = 109)	61% (n = 83)	p = 0.0095	V = 0.15
Hypertension	9.2% (n = 9)	10% (n = 25)	13% (n = 17)	ns (p = 0.82)	V = 0.041
Cardiovascular disease	0% (n = 0)	2.8% (n = 7)	2.2% (n = 3)	ns (p = 0.36)	V = 0.076
Diabetes	2% (n = 2)	1.6% (n = 4)	0.74% (n = 1)	ns (p = 0.82)	V = 0.04
Pulmonary disease	0% (n = 0)	4.5% (n = 11)	5.2% (n = 7)	ns (p = 0.14)	V = 0.1
Gastrointestinal disease	1% (n = 1)	2% (n = 5)	1.5% (n = 2)	ns (p = 0.88)	V = 0.032
Malignancy	0% (n = 0)	0.81% (n = 2)	5.9% (n = 8)	p = 0.0025	V = 0.17

Variable[a]	Cluster #1	Cluster #2	Cluster #3	Significance[b]	Effect size[b]
Hay fever/allergy	13% (n = 13)	17% (n = 41)	25% (n = 34)	ns (p = 0.073)	V = 0.12
Autoimmunity	7.1% (n = 7)	4.1% (n = 10)	11% (n = 15)	ns (p = 0.056)	V = 0.12
Freq. resp. infections	5.1% (n = 5)	5.3% (n = 13)	10% (n = 14)	ns (p = 0.2)	V = 0.093
Freq. bact. Infections	1% (n = 1)	3.7% (n = 9)	9.6% (n = 13)	p = 0.01	V = 0.15
Pre-CoV depression/anxiety	6.1% (n = 6)	2.8% (n = 7)	9.6% (n = 13)	p = 0.038	V = 0.13
Pre-CoV sleep disorders	5.1% (n = 5)	2.4% (n = 6)	4.4% (n = 6)	ns (p = 0.53)	V = 0.063
Daily medication	absent: 66% (n = 65)<br>1 - 4 drugs: 30% (n = 29)<br>5 drugs and more: 4.1% (n = 4)	absent: 66% (n = 162)<br>1 - 4 drugs: 33% (n = 80)<br>5 drugs and more: 1.6% (n = 4)	absent: 50% (n = 68)<br>1 - 4 drugs: 49% (n = 66)<br>5 drugs and more: 0.74% (n = 1)	p = 0.0092	V = 0.13

[a]BMI: body mass index, normal: BMI < 25 kg/m^2, overweight: BMI 25 - 30 kg/m^2, obesity: BMI > 30 kg/m^2; Pre-CoV depression/anxiety: depression or anxiety before COVID-19; Freq. resp. infections: frequent (> 2 per year) respiratory infections; Freq. bact. Infections: frequent (> two per year) bacterial infections with antibiotic therapy; Pre-CoV sleep disorders: sleep disorders before COVID-19.

[b]Categorical variables: χ^2 test with Cramer V effect size statistic. Numeric variables: Kruskal-Wallis test with η^2 effect size statistic. P values corrected form multiple testing with Benjamini-Hochberg method.

Supplementary Table S7: Demographic and baseline clinical characteristic at the COVID-19 onset of the survey study participants assigned to the recovery clusters, Italy (IT) cohort. Numeric variables are presented as medians with interquartile ranges (IQR) and ranges. Categorical variables are presented as percentages and counts within the complete observation set.

**Variable**[a]	**Cluster #1**	**Cluster #2**	**Cluster #3**	**Significance**[b]	**Effect size**[b]
Sex	female: 84% (n = 52)<br>male: 16% (n = 10)	female: 64% (n = 155)<br>male: 36% (n = 89)	female: 77% (n = 93)<br>male: 23% (n = 28)	p = 0.003	V = 0.18
Age, years	47 [IQR: 35 - 55]<br>range: 18 - 71	43 [IQR: 32 - 52]<br>range: 18 - 77	47 [IQR: 38 - 56]<br>range: 19 - 95	p = 0.024	η^2 = 0.016
BMI before COVID-19	normal: 82% (n = 51)<br>overweight: 11% (n = 7)<br>obesity: 6.5% (n = 4)	normal: 67% (n = 160)<br>overweight: 26% (n = 63)<br>obesity: 6.3% (n = 15)	normal: 56% (n = 67)<br>overweight: 29% (n = 34)<br>obesity: 15% (n = 18)	p = 0.0038	V = 0.14
Education	non-tertiary: 61% (n = 38)<br>tertiary: 39% (n = 24)	non-tertiary: 56% (n = 136)<br>tertiary: 44% (n = 108)	non-tertiary: 63% (n = 76)<br>tertiary: 37% (n = 45)	ns (p = 0.41)	V = 0.067
Employment status	employed: 81% (n = 50)<br>unemployed: 11% (n = 7)<br>leave: 1.6% (n = 1)<br>retired: 6.5% (n = 4)	employed: 80% (n = 194)<br>unemployed: 11% (n = 27)<br>leave: 2.9% (n = 7)<br>retired: 6.6% (n = 16)	employed: 86% (n = 104)<br>unemployed: 5% (n = 6)<br>leave: 0% (n = 0)<br>retired: 9.1% (n = 11)	ns (p = 0.3)	V = 0.098
Observation time	140 [IQR: 120 - 280]<br>range: 92 - 370	130 [IQR: 110 - 260]<br>range: 90 - 390	140 [IQR: 120 - 300]<br>range: 90 - 380	ns (p = 0.14)	η^2 = 0.0065
Comorbidity	37% (n = 23)	37% (n = 91)	59% (n = 71)	p < 0.001	V = 0.19
Hypertension	9.7% (n = 6)	6.6% (n = 16)	12% (n = 14)	ns (p = 0.33)	V = 0.081
Cardiovascular disease	0% (n = 0)	3.3% (n = 8)	4.1% (n = 5)	ns (p = 0.35)	V = 0.076
Diabetes	1.6% (n = 1)	0% (n = 0)	0% (n = 0)	ns (p = 0.085)	V = 0.12
Pulmonary disease	3.2% (n = 2)	2% (n = 5)	4.1% (n = 5)	ns (p = 0.51)	V = 0.056
Gastrointestinal disease	0% (n = 0)	0.41% (n = 1)	1.7% (n = 2)	ns (p = 0.36)	V = 0.073
Malignancy	8.1% (n = 5)	2.5% (n = 6)	5% (n = 6)	ns (p = 0.15)	V = 0.1

Variable[a]	Cluster #1	Cluster #2	Cluster #3	Significance[b]	Effect size[b]
Hay fever/allergy	8.1% (n = 5)	11% (n = 28)	15% (n = 18)	ns (p = 0.41)	V = 0.067
Autoimmunity	6.5% (n = 4)	4.9% (n = 12)	9.1% (n = 11)	ns (p = 0.35)	V = 0.075
Freq. resp. infections	0% (n = 0)	1.2% (n = 3)	9.1% (n = 11)	p < 0.001	V = 0.21
Freq. bact. Infections	0% (n = 0)	0.41% (n = 1)	3.3% (n = 4)	ns (p = 0.058)	V = 0.13
Pre-CoV depression/anxiety	3.2% (n = 2)	2.9% (n = 7)	11% (n = 13)	p = 0.009	V = 0.16
Pre-CoV sleep disorders	3.2% (n = 2)	2% (n = 5)	11% (n = 13)	p = 0.0022	V = 0.18
Daily medication	absent: 73% (n = 45)<br>1 - 4 drugs: 27% (n = 17)<br>5 drugs and more: 0% (n = 0)	absent: 81% (n = 197)<br>1 - 4 drugs: 19% (n = 46)<br>5 drugs and more: 0.41% (n = 1)	absent: 62% (n = 75)<br>1 - 4 drugs: 36% (n = 43)<br>5 drugs and more: 2.5% (n = 3)	p = 0.0034	V = 0.14

[a]BMI: body mass index, normal: BMI < 25 kg/m^2, overweight: BMI 25 - 30 kg/m^2, obesity: BMI > 30 kg/m^2; Pre-CoV depression/anxiety: depression or anxiety before COVID-19; Freq. resp. infections: frequent (> 2 per year) respiratory infections; ;Freq. bact. Infections: frequent (> two per year) bacterial infections with antibiotic therapy; Pre-CoV sleep disorders: sleep disorders before COVID-19.

[b]Categorical variables: χ^2 test with Cramer V effect size statistic. Numeric variables: Kruskal-Wallis test with η^2 effect size statistic. P values corrected form multiple testing with Benjamini-Hochberg method.

Supplementary Table S8: COVID-19 course and recovery in the survey study participants assigned to the recovery clusters, Austria (AT) cohort. Numeric variables are presented as medians with interquartile ranges (IQR) and ranges. Categorical variables are presented as percentages and counts within the complete observation set.

Variable[a]	Cluster #1	Cluster #2	Cluster #3	Significance[a]	Effect size[a]
SARS-CoV2 outbreak	spring 2020: 55% (n = 54)<br>summer/fall 2020: 43% (n = 42)<br>winter/spring 2021: 2% (n = 2)	spring 2020: 63% (n = 156)<br>summer/fall 2020: 35% (n = 87)<br>winter/spring 2021: 1.2% (n = 3)	spring 2020: 53% (n = 71)<br>summer/fall 2020: 47% (n = 64)<br>winter/spring 2021: 0% (n = 0)	ns (p = 0.16)	V = 0.09
Weight loss, kg	0.5 [IQR: 0 - 3]<br>range: 0 - 8	0 [IQR: 0 - 2.1]<br>range: 0 - 11	2 [IQR: 0 - 4.5]<br>range: 0 - 15	$p < 0.001$	$\eta^2 = 0.039$
Hair loss	19% (n = 19)	9.3% (n = 23)	30% (n = 41)	$p < 0.001$	V = 0.24
Incomplete recovery	62% (n = 61)	22% (n = 55)	73% (n = 98)	$p < 0.001$	V = 0.47
Physical performance loss, percent	10 [IQR: 4 - 25]<br>range: 0 - 69	3.5 [IQR: 0 - 14]<br>range: 0 - 100	25 [IQR: 15 - 42]<br>range: 0 - 92	$p < 0.001$	$\eta^2 = 0.26$
New medication after COVID-19	7.1% (n = 7)	7.3% (n = 18)	24% (n = 32)	$p < 0.001$	V = 0.23
Subjective need for rehabilitation	13% (n = 13)	6.5% (n = 16)	42% (n = 56)	$p < 0.001$	V = 0.4
ANX score	0 [IQR: 0 - 2]<br>range: 0 - 5	0 [IQR: 0 - 1]<br>range: 0 - 6	1.5 [IQR: 0 - 2]<br>range: 0 - 6	$p < 0.001$	$\eta^2 = 0.11$
DPR score	1 [IQR: 0 - 2]<br>range: 0 - 6	0 [IQR: 0 - 2]<br>range: 0 - 6	2 [IQR: 1 - 3]<br>range: 0 - 6	$p < 0.001$	$\eta^2 = 0.15$
Stress score	3.5 [IQR: 2 - 6]<br>range: 0 - 19	3 [IQR: 1 - 5]<br>range: 0 - 16	5 [IQR: 3 - 9]<br>range: 0 - 16	$p < 0.001$	$\eta^2 = 0.064$
OMH impairment score	1 [IQR: 0 - 1]<br>range: 0 - 3	1 [IQR: 0 - 1]<br>range: 0 - 3	1 [IQR: 1 - 2]<br>range: 0 - 3	$p < 0.001$	$\eta^2 = 0.072$
QoL impairment score	1 [IQR: 1 - 1]<br>range: 0 - 3	1 [IQR: 0 - 1]<br>range: 0 - 3	1 [IQR: 1 - 2]<br>range: 0 - 3	$p < 0.001$	$\eta^2 = 0.052$

[a]Incomplete recovery: self-reported incomplete recovery from COVID-19; Physical performance loss: self-rated physical performance loss after COVID-19, before COVID-19: 100%; ANX score: anxiety score, Patient Health Questionnaire, PHQ-4; DPR: depression score, Patient Health Questionnaire, PHQ-4; Stress score: mental stress score; 7 item PHQ stress module; OMH impairment score: score of overall mental health impairment; QoL impairment score: score of impaired quality of life.

Variable[a]	Cluster #1	Cluster #2	Cluster #3	Significance[a]	Effect size[a]

[a]Categorical variables: χ^2 test with Cramer V effect size statistic. Numeric variables: Kruskal-Wallis test with η^2 effect size statistic. P values corrected form multiple testing with Benjamini-Hochberg method.

Supplementary Table S9: COVID-19 course and recovery in the survey study participants assigned to the recovery clusters, Italy (IT) cohort. Numeric variables are presented as medians with interquartile ranges (IQR) and ranges. Categorical variables are presented as percentages and counts within the complete observation set.

**Variable**[a]	**Cluster #1**	**Cluster #2**	**Cluster #3**	**Significance**[a]	**Effect size**[a]
SARS-CoV2 outbreak	spring 2020: 35% (n = 22)<br>summer/fall 2020: 63% (n = 39)<br>winter/spring 2021: 1.6% (n = 1)	spring 2020: 28% (n = 68)<br>summer/fall 2020: 72% (n = 175)<br>winter/spring 2021: 0.41% (n = 1)	spring 2020: 32% (n = 39)<br>summer/fall 2020: 68% (n = 82)<br>winter/spring 2021: 0% (n = 0)	ns (p = 0.41)	V = 0.069
Weight loss, kg	0 [IQR: 0 - 2]<br>range: 0 - 5	0 [IQR: 0 - 2]<br>range: 0 - 8	2 [IQR: 0 - 4]<br>range: 0 - 15	p < 0.001	$\eta^2 = 0.046$
Hair loss	27% (n = 17)	10% (n = 25)	30% (n = 36)	p < 0.001	V = 0.24
Incomplete recovery	56% (n = 34)	18% (n = 44)	67% (n = 81)	p < 0.001	V = 0.47
Physical performance loss, percent	10 [IQR: 1 - 21]<br>range: 0 - 90	5 [IQR: 0 - 17]<br>range: 0 - 60	30 [IQR: 20 - 50]<br>range: 0 - 93	p < 0.001	$\eta^2 = 0.3$
New medication after COVID-19	15% (n = 9)	8.4% (n = 20)	19% (n = 23)	p = 0.023	V = 0.15
Subjective need for rehabilitation	18% (n = 11)	3.7% (n = 9)	35% (n = 42)	p < 0.001	V = 0.39
ANX score	1 [IQR: 0 - 2]<br>range: 0 - 6	0 [IQR: 0 - 2]<br>range: 0 - 6	2 [IQR: 1 - 4]<br>range: 0 - 6	p < 0.001	$\eta^2 = 0.14$
DPR score	2 [IQR: 0 - 2]<br>range: 0 - 6	1 [IQR: 0 - 2]<br>range: 0 - 6	2 [IQR: 2 - 4]<br>range: 0 - 6	p < 0.001	$\eta^2 = 0.16$
Stress score	4 [IQR: 2 - 6.8]<br>range: 0 - 13	3 [IQR: 1 - 6]<br>range: 0 - 14	6 [IQR: 4 - 8]<br>range: 0 - 15	p < 0.001	$\eta^2 = 0.11$
OMH impairment score	1 [IQR: 0 - 1]<br>range: 0 - 3	1 [IQR: 0 - 1]<br>range: 0 - 3	1 [IQR: 1 - 2]<br>range: 0 - 3	p < 0.001	$\eta^2 = 0.099$
QoL impairment score	1 [IQR: 1 - 1.8]<br>range: 0 - 3	1 [IQR: 0 - 1]<br>range: 0 - 3	1 [IQR: 1 - 2]<br>range: 0 - 3	p < 0.001	$\eta^2 = 0.1$

[a]Incomplete recovery: self-reported incomplete recovery from COVID-19; Physical performance loss: self-rated physical performance loss after COVID-19, before COVID-19: 100%; ANX score: anxiety score, Patient Health Questionnaire, PHQ-4; DPR: depression score, Patient Health Questionnaire, PHQ-4; Stress score: mental stress score; 7 item PHQ stress module; OMH impairment score: score of overall mental health impairment; QoL impairment score: score of impaired quality of life.

Variable[a]	Cluster #1	Cluster #2	Cluster #3	Significance[a]	Effect size[a]

[a]Categorical variables: χ^2 test with Cramer V effect size statistic. Numeric variables: Kruskal-Wallis test with η^2 effect size statistic. P values corrected form multiple testing with Benjamini-Hochberg method.

Supplementary Figures

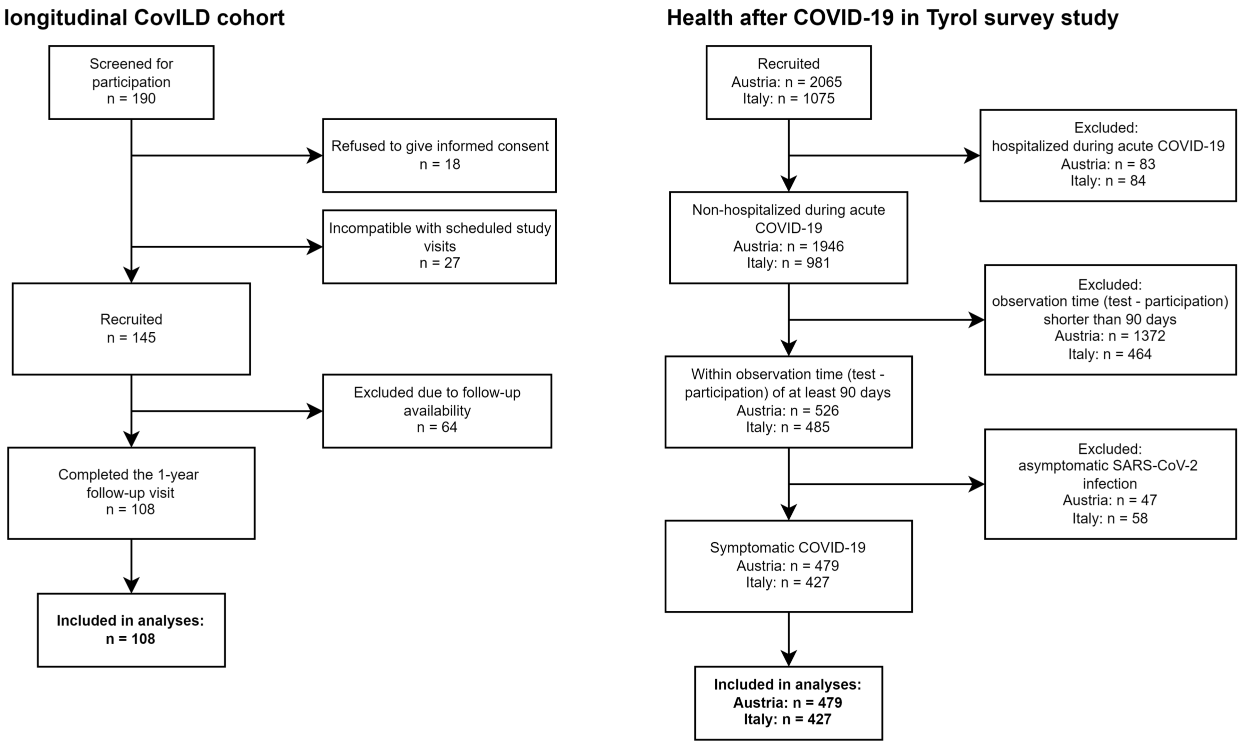

Supplementary Figure S1. Flow diagram of the analysis inclusion process for the observational CovILD cohort and the Health after COVID-19 survey study.

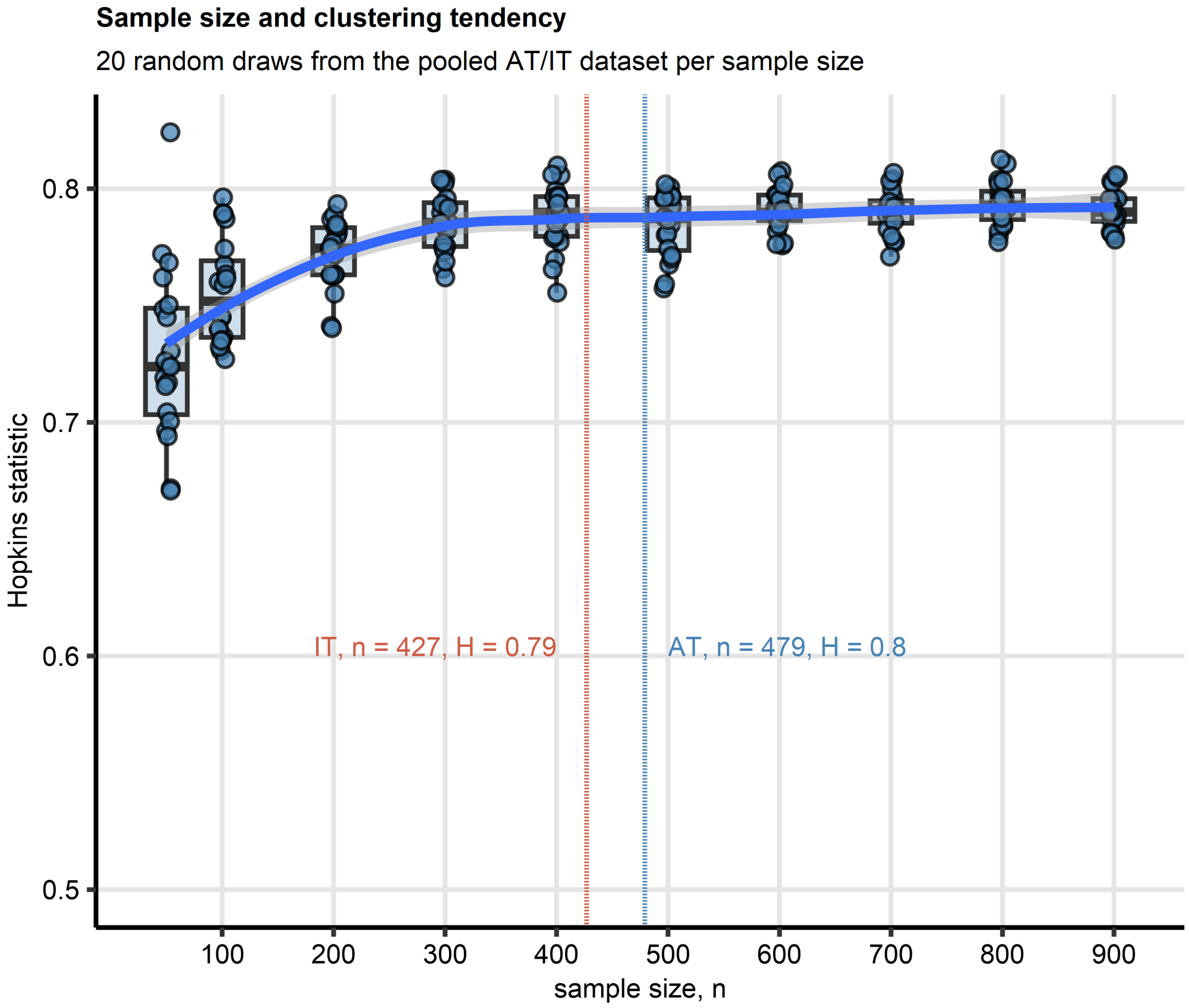

Supplementary Figure S2. Estimation of sample size for clustering analysis with the survey study datasets.

To assess the minimal sample size of the survey study for clustering, random subsets of the pooled Austria (AT) and Italy (IT) COVID-19 symptom data set with differing observation numbers (50, 100, 200, 300, 400, 500, 600, 700, 800, 900 observations, 20 random subsets [draws] per sample size) were generated and their clustering tendency was assessed with Hopkins statistic (H). Median H values per sample size with interquartile ranges (IQR) are visualized as boxes. Whiskers span over the 150% IQR. Single H values are depicted as points. Blue line with gray ribbon represents the LOESS trend with 95% confidence interval. Dashed lines represent sample sizes of the AT (blue) and IT cohort (blue). Sample sizes and H values for the AT and IT collectives are displayed in the plot. Note: beginning from the sample size of n = 400, no improvement of the clustering tendency could be observed. This suggest n = 400 as minimal sample size required for reproducible clustering analysis results.

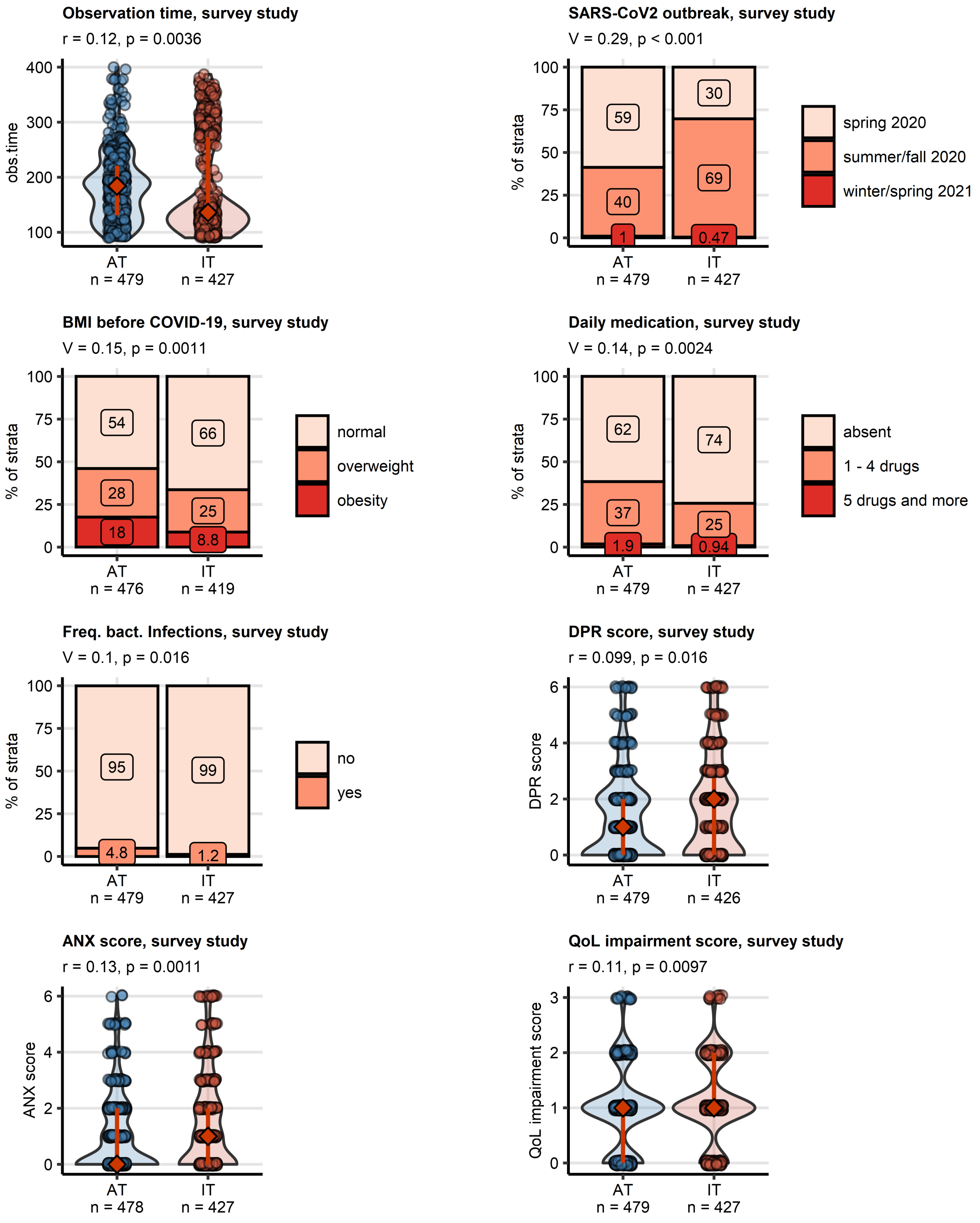
Observation time, survey study
r = 0.12, p = 0.0036
obs.time
AT n = 479
IT n = 427
SARS-CoV2 outbreak, survey study
V = 0.29, p < 0.001
% of strata
spring 2020
summer/fall 2020
winter/spring 2021
AT n = 479
IT n = 427
BMI before COVID-19, survey study
V = 0.15, p = 0.0011
% of strata
normal
overweight
obesity
AT n = 476
IT n = 419
Daily medication, survey study
V = 0.14, p = 0.0024
% of strata
absent
1 - 4 drugs
5 drugs and more
AT n = 479
IT n = 427
Freq. bact. Infections, survey study
V = 0.1, p = 0.016
% of strata
no
yes
AT n = 479
IT n = 427
DPR score, survey study
r = 0.099, p = 0.016
DPR score
AT n = 479
IT n = 426
ANX score, survey study
r = 0.13, p = 0.0011
ANX score
AT n = 478
IT n = 427
QoL impairment score, survey study
r = 0.11, p = 0.0097
QoL impairment score
AT n = 479
IT n = 427

Supplementary Figure S3. The largest significant differences in demographic, clinical and recovery variables between the Austria and Italy cohorts of the survey study.

Differences in numeric variables between the Austria (AT) and Italy (IT) survey study cohorts were assessed by Mann-Whitney test with r effect size statistic. Differences in categorical variables were investigated by χ^2 *test with Cramer's V effect size statistic. P values were corrected for multiple testing with Benjamini-Hochberg method. Significant numeric variables are presented in violin plots with medians and interquartile ranges depicted as diamonds and whiskers and single observations visualized as points. Percentages of categories of qualitative variables within the AT and IT cohorts are displayed as stack plots. Effect sizes and p values are presented in the plot captions. Numbers of complete observations are indicated in the plot axes.*

BMI before COVID-19: body mass index before COVID-19, normal: BMI < 25 kg/m0B2, overweight: BMI 25 - 30 kg/m0B2, obesity: BMI > 30 kg/0B2; Freq. bact. Infections: frequent (> 2 per year) bacterial infection requiring an antibiotic treatment; DPR score: depression score, Patient Health Questionnaire, PHQ-4; ANX score: anxiety score, Patient Health Questionnaire, PHQ-4; QoL impairment score: score of impairment of quality of life.

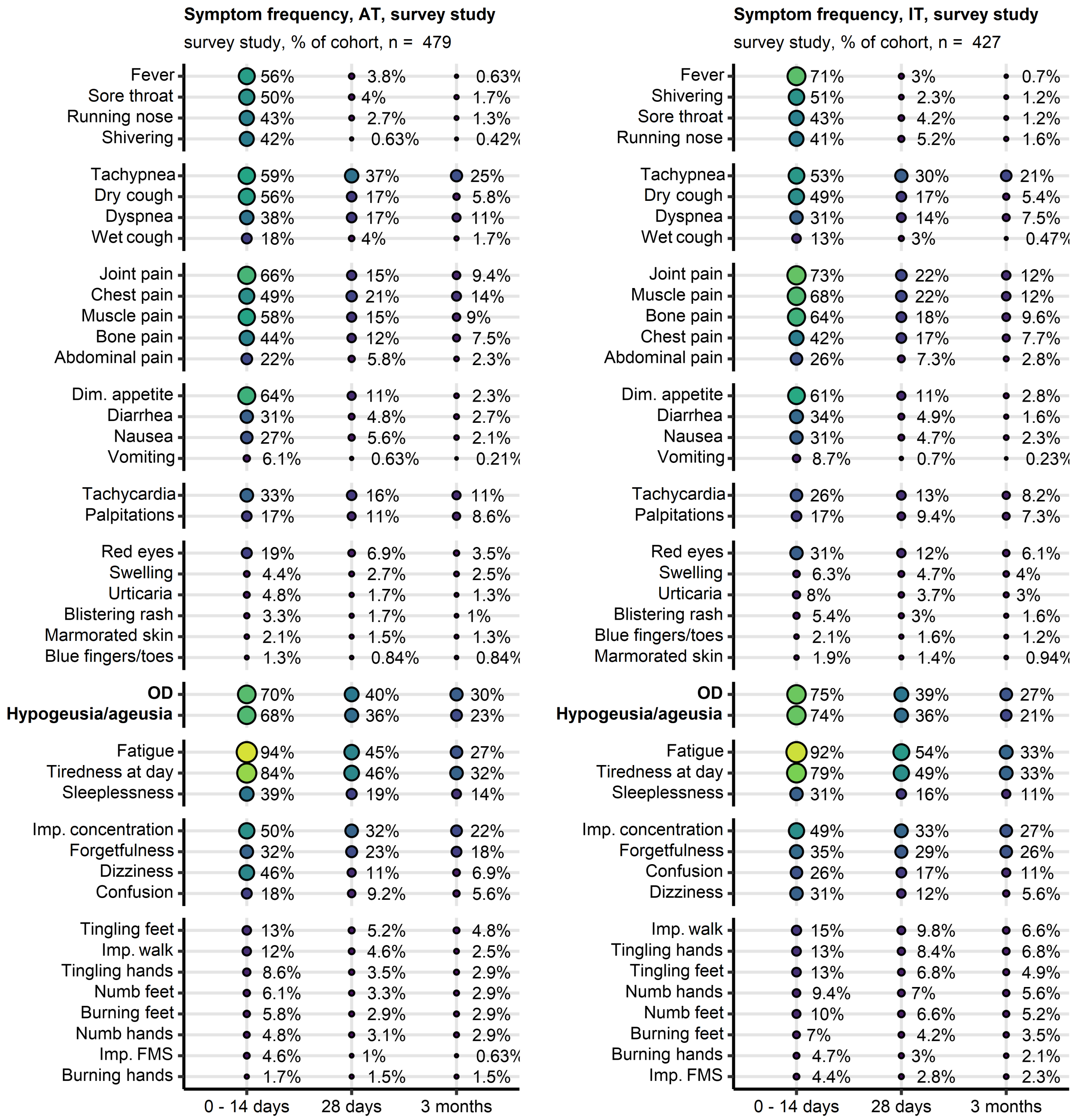

Supplementary Figure S4. Frequency of COVID-19 symptoms in the survey study.

Frequency of symptoms in first 14 days, at 28 days and at three months after clinical onset of COVID-19 in the Austria (AT) and Italy (IT) survey study cohorts expressed as percentages of the cohort. Point size and color represents the percentage. Numbers of complete observations are indicated in the plot captions.

OD: self-reported olfactory dysfunction; Dim. appetite: diminished appetite; Imp. concentration: impaired concentration; Imp. walk: impaired walk; Imp. FMS: impaired fine motor skills.

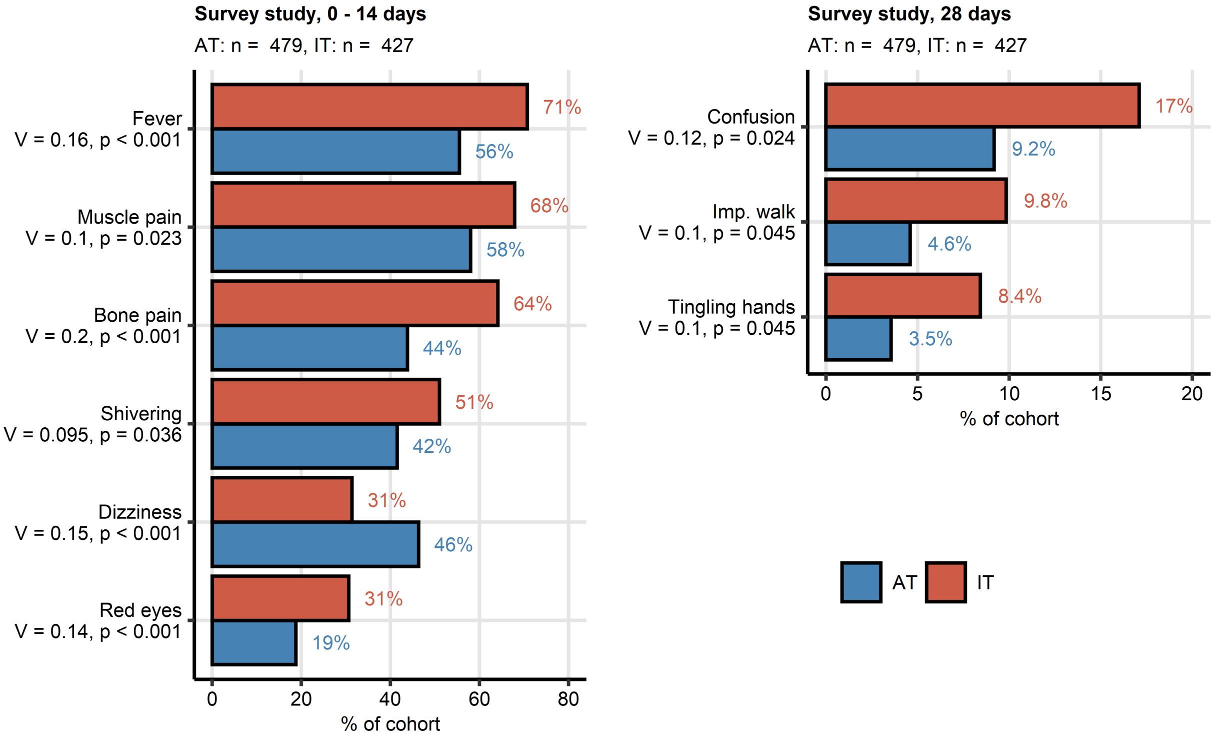

Supplementary Figure S5. Significant differences in frequency of COVID-19 symptoms in the survey study.

Differences in frequency of COVID-19-related symptoms between the Austria (AT) and Italy (IT) cohorts of the survey study in the first 14 days, at 28 days and at 3 months after clinical onset were assessed by χ^2 test with Cramer's V effect size statistic. P values were adjusted for multiple testing with Benjamini-Hochberg method. Significant effects for the 14 and 28 day time points are plotted. No significant differences in symptom frequency at the 3-month time point could be observed. Symptom percentages within the cohort are displayed in bar plots. Effect sizes and p values are displayed in the Y axes. Numbers of complete observations are shown in the plot captions.

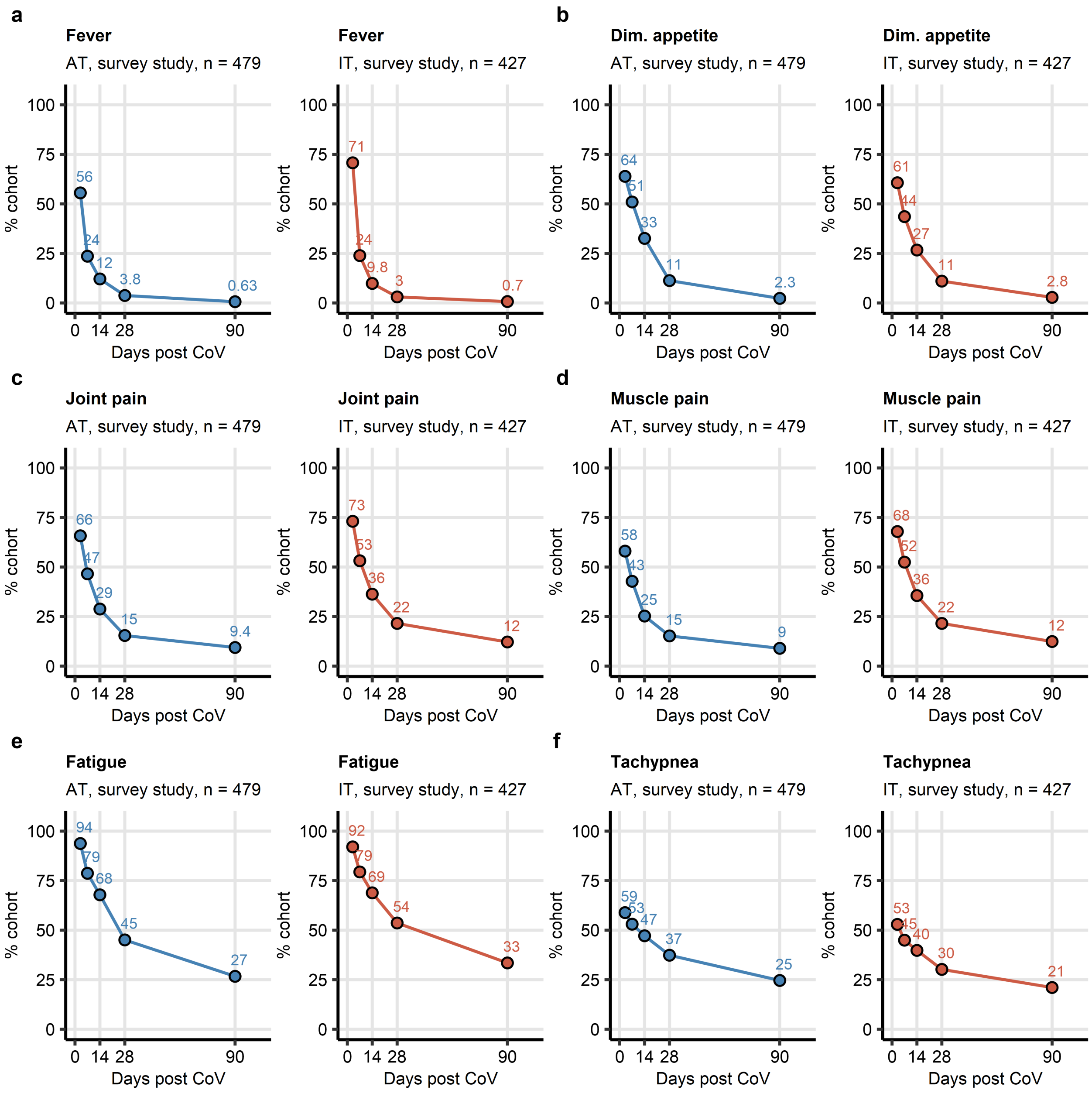

Supplementary Figure S6. Kinetic of recovery from leading acute COVID-19 symptoms in the survey study.

Percentages of individuals with fever (a), diminished appetite (b), joint pain (c), muscle pain (d), fatigue (e) and tachypnea (f) in the AT (Austria) and IT (Italy) survey study cohorts at particular time points after clinical onset. Numbers of complete observations are indicated under the plots.

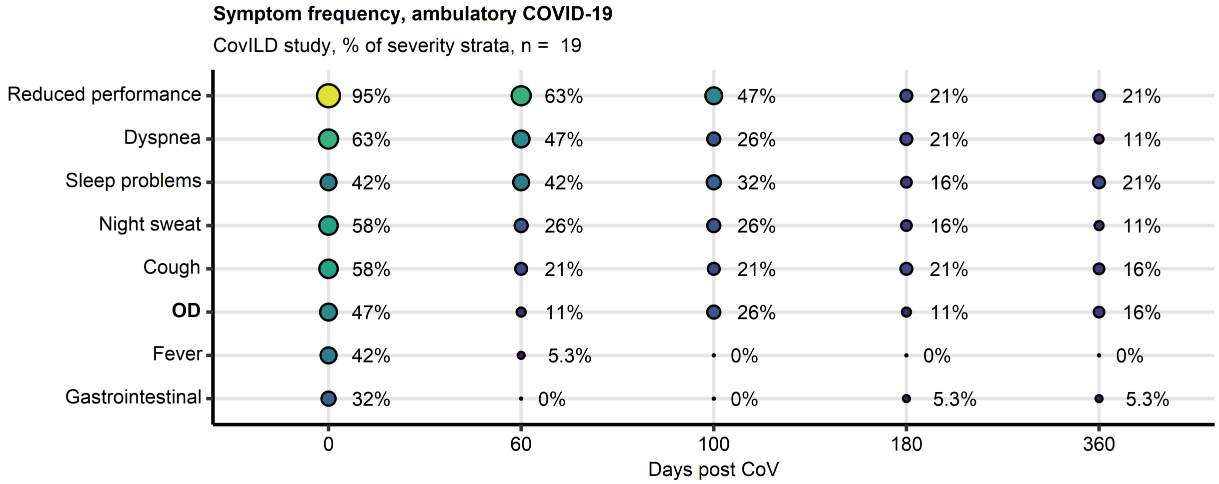
Symptom frequency, ambulatory COVID-19
CovILD study, % of severity strata, n = 19
Reduced performance
Dyspnea
Sleep problems
Night sweat
Cough
OD
Fever
Gastrointestinal
95%
63%
42%
58%
58%
47%
42%
32%
63%
47%
42%
26%
21%
11%
5.3%
0%
47%
26%
32%
26%
21%
26%
0%
0%
21%
21%
16%
16%
21%
11%
0%
5.3%
21%
11%
21%
11%
16%
16%
0%
5.3%
0
60
100
180
360
Days post CoV

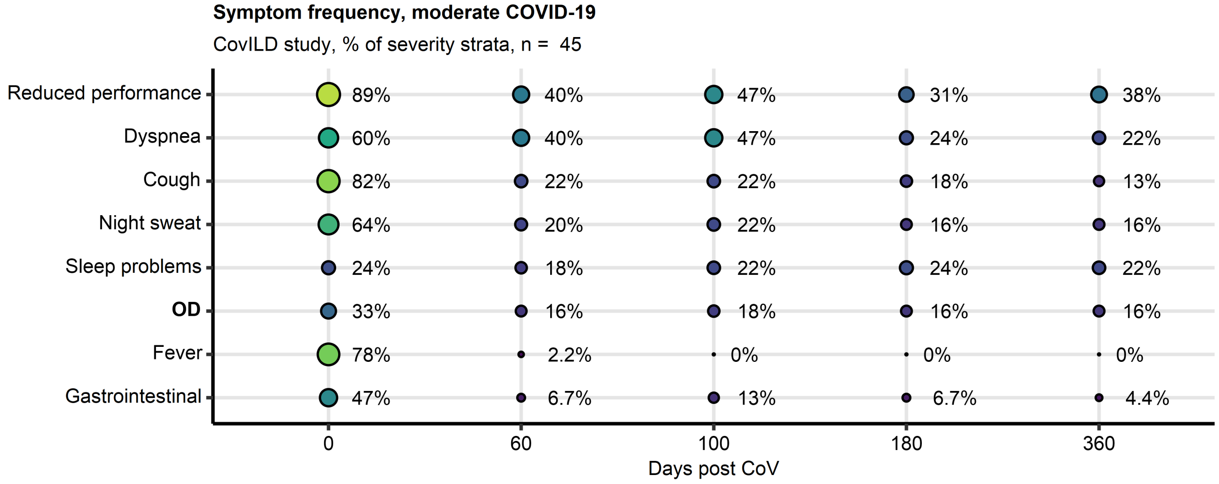
Symptom frequency, moderate COVID-19
CovILD study, % of severity strata, n = 45
Reduced performance
Dyspnea
Cough
Night sweat
Sleep problems
OD
Fever
Gastrointestinal
89%
60%
82%
64%
24%
33%
78%
47%
40%
40%
22%
20%
18%
16%
2.2%
6.7%
47%
47%
22%
22%
22%
18%
0%
13%
31%
24%
18%
16%
24%
16%
0%
6.7%
38%
22%
13%
16%
22%
16%
0%
4.4%
0
60
100
180
360
Days post CoV

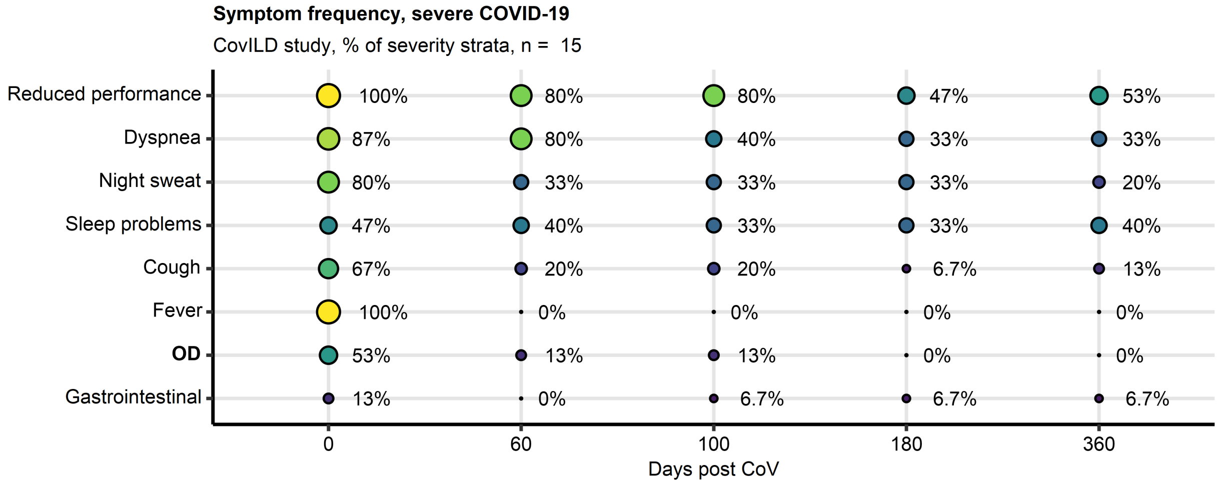
Symptom frequency, severe COVID-19
CovILD study, % of severity strata, n = 15
Reduced performance
Dyspnea
Night sweat
Sleep problems
Cough
Fever
OD
Gastrointestinal
100%
87%
80%
47%
67%
100%
53%
13%
80%
80%
33%
40%
20%
0%
13%
0%
80%
40%
33%
33%
20%
0%
13%
6.7%
47%
33%
33%
33%
6.7%
0%
0%
6.7%
53%
33%
20%
40%
13%
0%
0%
6.7%
0
60
100
180
360
Days post CoV

Supplementary Figure S7. Symptom frequency in ambulatory, moderate and severe COVID-19 subsets of the CovILD study.

Frequency of symptoms during acute COVID-19 and at the 60-, 100-, 180- and 360-day follow-ups in ambulatory, moderate and severe COVID-19 participants expressed as percentages of individuals with the complete longitudinal data set. Point size and color represents the percentage. Numbers of complete observations are indicated under the plots.

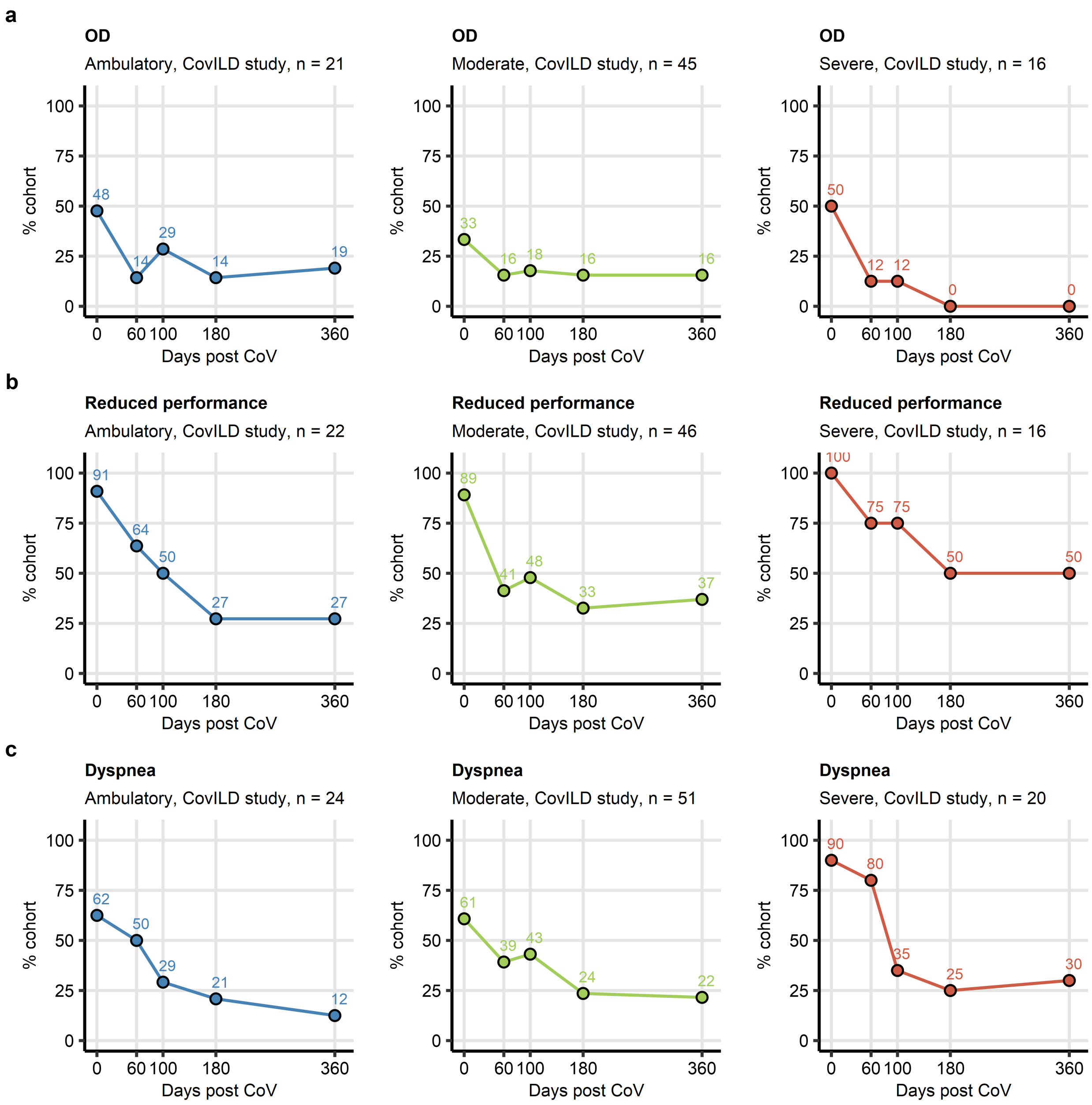

Supplementary Figure S8. Kinetic of recovery from olfactory dysfunction, reduced performance and dyspnea in ambulatory, moderate and severe COVID-19 subsets of the CovILD study.

Percentages of individuals with the complete longitudinal data set suffering from olfactory dysfunction (OD) (a), reduced physical performance (b) and dyspnea (c) in the ambulatory, moderate and severe COVID-19 subsets during acute COVID-19 and at the 60-, 100-, 180- and 360-day follow-ups. Numbers of complete observations are indicated under the plots.

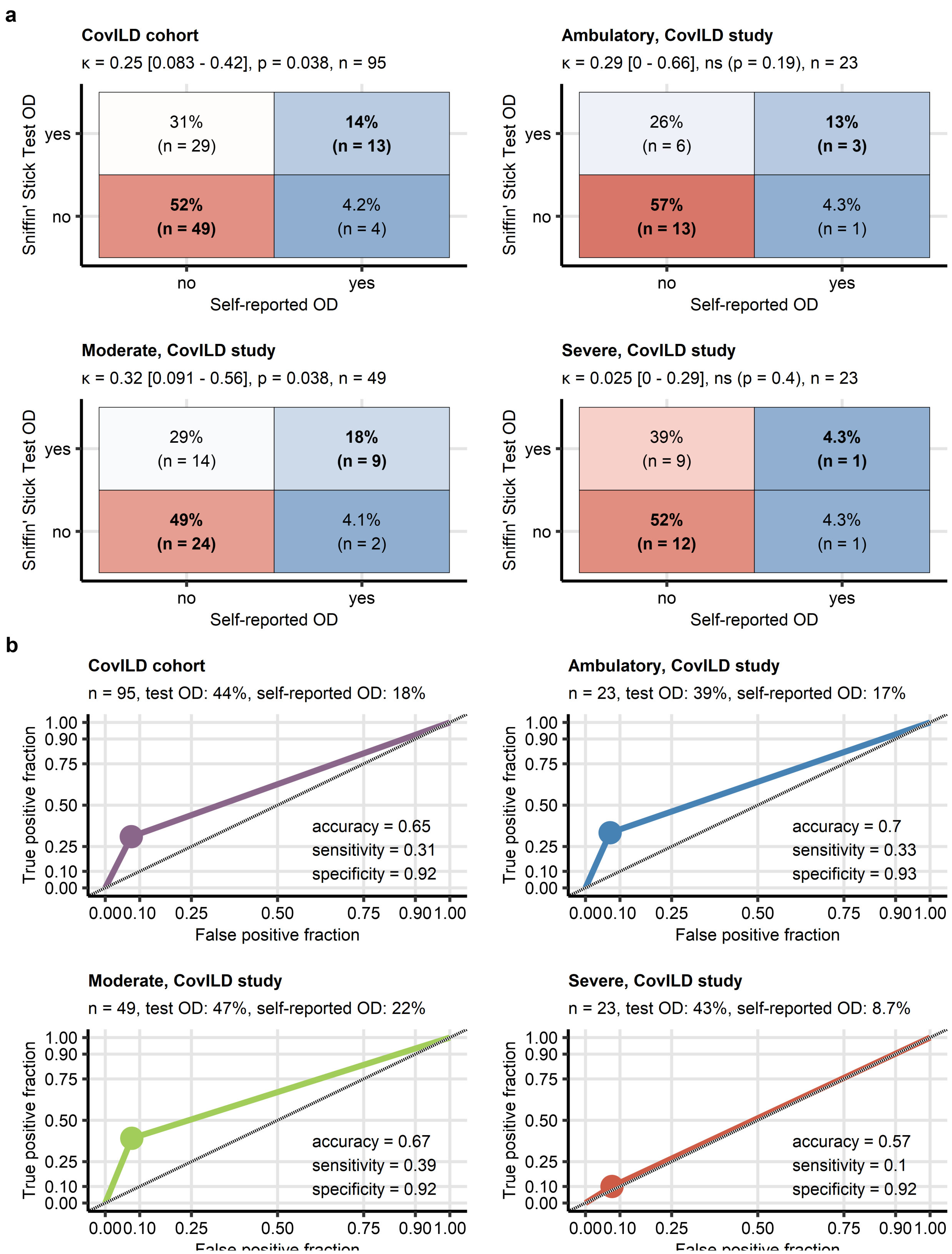
a
CovILD cohort
κ = 0.25 [0.083 - 0.42], p = 0.038, n = 95
31% (n = 29)
14% (n = 13)
52% (n = 49)
4.2% (n = 4)
Ambulatory, CovILD study
κ = 0.29 [0 - 0.66], ns (p = 0.19), n = 23
26% (n = 6)
13% (n = 3)
57% (n = 13)
4.3% (n = 1)
Moderate, CovILD study
κ = 0.32 [0.091 - 0.56], p = 0.038, n = 49
29% (n = 14)
18% (n = 9)
49% (n = 24)
4.1% (n = 2)
Severe, CovILD study
κ = 0.025 [0 - 0.29], ns (p = 0.4), n = 23
39% (n = 9)
4.3% (n = 1)
52% (n = 12)
4.3% (n = 1)
Sniffin' Stick Test OD
Self-reported OD
yes
no
b
CovILD cohort
n = 95, test OD: 44%, self-reported OD: 18%
accuracy = 0.65
sensitivity = 0.31
specificity = 0.92
Ambulatory, CovILD study
n = 23, test OD: 39%, self-reported OD: 17%
accuracy = 0.7
sensitivity = 0.33
specificity = 0.93
Moderate, CovILD study
n = 49, test OD: 47%, self-reported OD: 22%
accuracy = 0.67
sensitivity = 0.39
specificity = 0.92
Severe, CovILD study
n = 23, test OD: 43%, self-reported OD: 8.7%
accuracy = 0.57
sensitivity = 0.1
specificity = 0.92
True positive fraction
False positive fraction
0.00
0.10
0.25
0.50
0.75
0.90
1.00

Supplementary Figure S9. Rates of self-reported olfactory dysfunction and olfactory dysfunction in the Sniffin' Stick Test at 3-month post COVID-19 follow-up in the ambulatory, moderate and severe COVID-19 subsets of the CovILD study.

Objective olfactory dysfunction (OD) was diagnosed in CovILD study participants with < 13 correctly identified odorants in the 16-item Sniffin' Sticks Identification Test. Frequencies of objective and self-reported OD were compared at the two-month follow-up after COVID-19 in the entire cohort and the ambulatory, hospitalized moderate COVID-19 and hospitalized severe COVID-19 patients.

(a) Rates of objective (test) and self-reported OD presented in heat maps of confusion matrices. The overall concordance between the objective and subjective OD was assessed with Cohen's κ inter-rater reliability statistic. Significance of κ was determined by Wald's Z test corrected for multiple testing with Benjamini-Hochberg method. κ values with 95% confidence intervals, p values and numbers of complete observations are displayed in the plot captions.

(b) Reliability of detection of objective OD by self-reported OD was assessed by receiver-operating characteristic (ROC). Sensitivity and specificity was visualized as ROC curves. numbers of complete observations, percentages of objective (test) and self-reported OD are displayed in the plot captions. Overall accuracy, sensitivity and specificity were are shown in the plots.

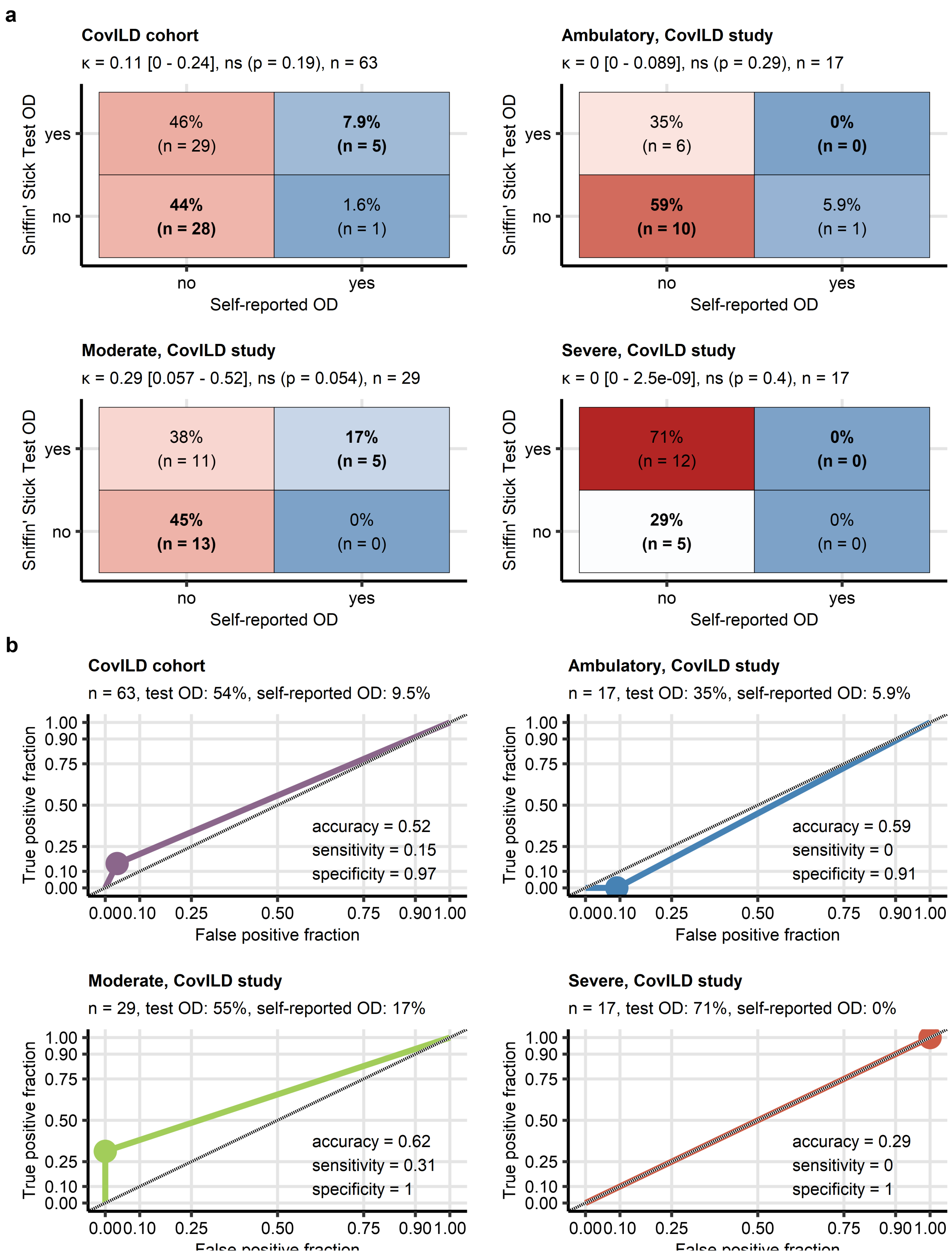
a
CovILD cohort
κ = 0.11 [0 - 0.24], ns (p = 0.19), n = 63
46% (n = 29)
7.9% (n = 5)
44% (n = 28)
1.6% (n = 1)
Ambulatory, CovILD study
κ = 0 [0 - 0.089], ns (p = 0.29), n = 17
35% (n = 6)
0% (n = 0)
59% (n = 10)
5.9% (n = 1)
Moderate, CovILD study
κ = 0.29 [0.057 - 0.52], ns (p = 0.054), n = 29
38% (n = 11)
17% (n = 5)
45% (n = 13)
0% (n = 0)
Severe, CovILD study
κ = 0 [0 - 2.5e-09], ns (p = 0.4), n = 17
71% (n = 12)
0% (n = 0)
29% (n = 5)
0% (n = 0)
Sniffin' Stick Test OD
yes
no
Self-reported OD
b
CovILD cohort
n = 63, test OD: 54%, self-reported OD: 9.5%
accuracy = 0.52
sensitivity = 0.15
specificity = 0.97
Ambulatory, CovILD study
n = 17, test OD: 35%, self-reported OD: 5.9%
accuracy = 0.59
sensitivity = 0
specificity = 0.91
Moderate, CovILD study
n = 29, test OD: 55%, self-reported OD: 17%
accuracy = 0.62
sensitivity = 0.31
specificity = 1
Severe, CovILD study
n = 17, test OD: 71%, self-reported OD: 0%
accuracy = 0.29
sensitivity = 0
specificity = 1
True positive fraction
False positive fraction
0.00 0.10 0.25 0.50 0.75 0.90 1.00

Supplementary Figure S10. Rates of self-reported olfactory dysfunction and olfactory dysfunction in the Sniffin' Stick Test at 1-year post COVID-19 follow-up in the ambulatory, moderate and severe COVID-19 subsets of the CovILD study.

Objective olfactory dysfunction (OD) was diagnosed in CovILD study participants with < 13 correctly identified odorants in the 16-item Sniffin' Sticks Identification Test. Frequencies of objective and self-reported OD were compared at the one-year follow-up after COVID-19 in the entire cohort and the ambulatory, hospitalized moderate COVID-19 and hospitalized severe COVID-19 patients.

(a) Rates of objective (test) and self-reported OD presented in heat maps of confusion matrices. The overall concordance between the objective and subjective OD was assessed with Cohen's κ inter-rater reliability statistic. Significance of κ was determined by Wald's Z test corrected for multiple testing with Benjamini-Hochberg method. κ values with 95% confidence intervals, p values and numbers of complete observations are displayed in the plot captions.

(b) Reliability of detection of objective OD by self-reported OD was assessed by receiver-operating characteristic (ROC). Sensitivity and specificity was visualized as ROC curves. numbers of complete observations, percentages of objective (test) and self-reported OD are displayed in the plot captions. Overall accuracy, sensitivity and specificity were are shown in the plots.

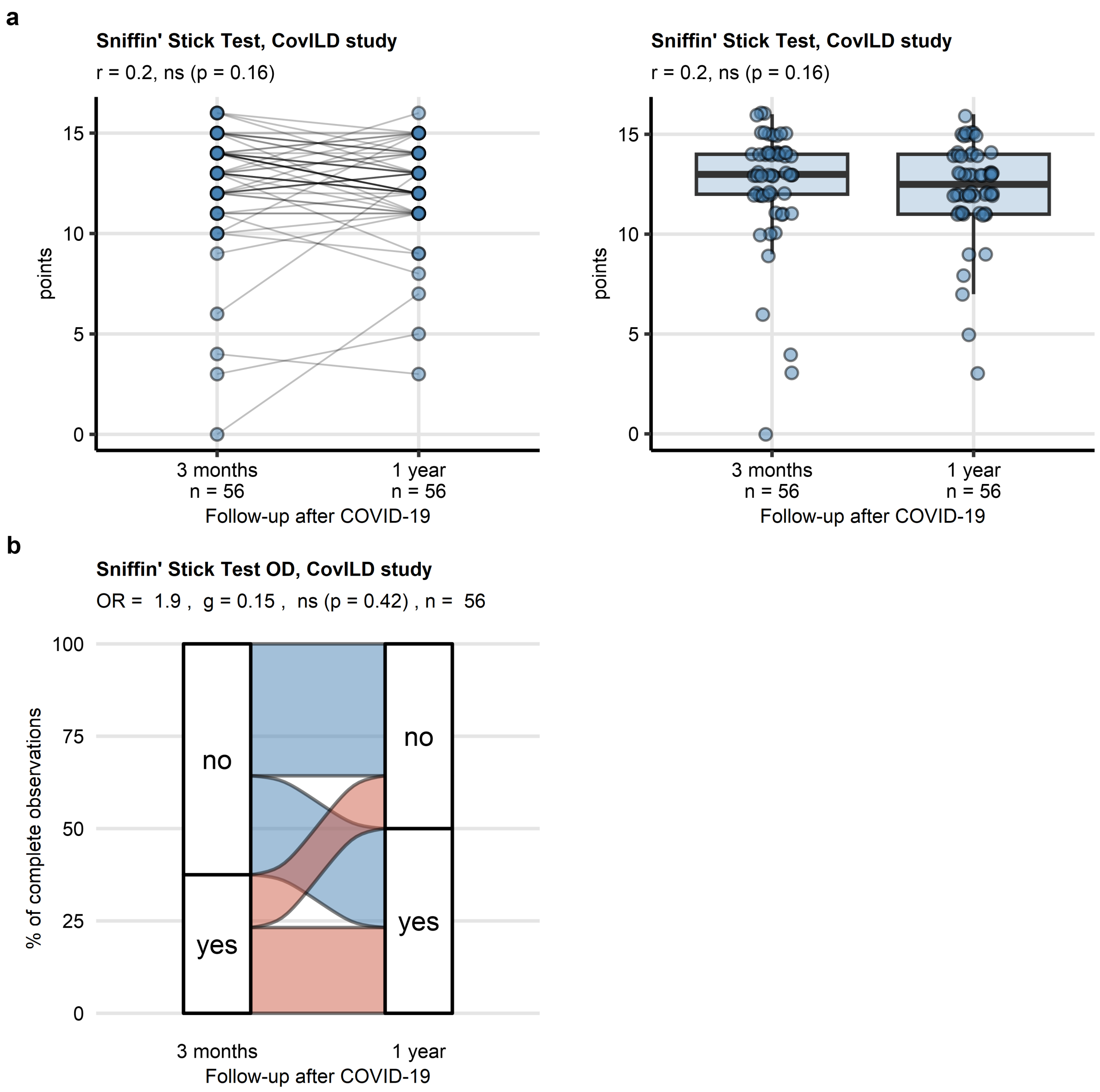

Supplementary Figure S11. Individuals trajectories of objective olfactory dysfunction in the CovILD study subset with the complete longitudinal follow-up data.

Objective olfactory dysfunction (OD) assessed in CovILD study participants with the Sniffin' Stick Identification test at the three-month and one-year follow-up after COVID-19. Objective olfactory dysfunction (OD) was diagnosed for < 13 correctly identified odorants in the 16-item Sniffin' Sticks Identification Test. A subset of the CovILD study participant with the complete Sniffin' Stick Test data for both follow-ups was analyzed.

(a) Comparison of numeric Sniffin' Stick Test results was done with paired Wilcoxon test with r effect size statistic. Results are presented as a before - after plot (left) and box plot (right). Medians with interquartile ranges (IQR) are visualized as boxes, whiskers span over 150% IQR. Single observations are depicted as points. Observations obtained for the same participant are connected with a line. Effect sizes, p values are displayed in the plot caption. Numbers of complete observations are indicated i the X axis.

(b) Comparison of frequencies of objective OD expressed as paired proportions was done with McNemar test with Cohen's q effect size statistic. Percentages of participants with objective OD at the three-month and one-year follow-up are displayed in an alluvial plot. The odds ratio of objective OD (one-year vs three months), effect size, p value and the number of participants are displayed in the plot caption.

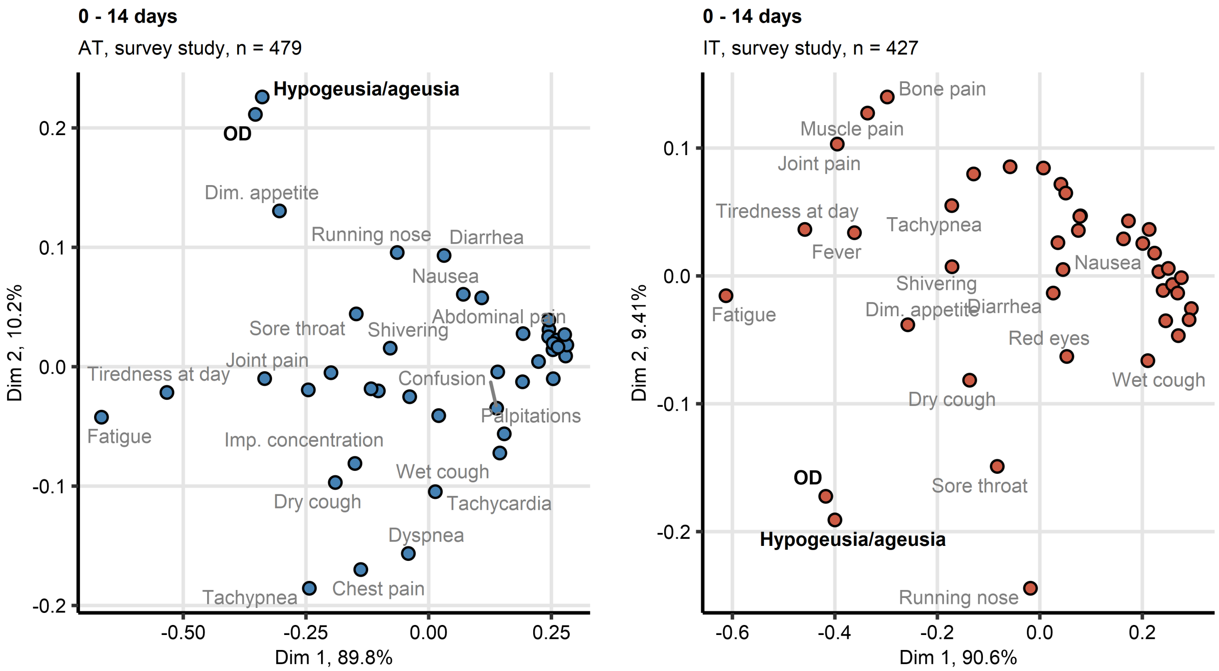

Supplementary Figure S12. Multi-dimensional scaling analysis of acute COVID-19 symptoms in the survey study.

Symptom data for acute COVID-19 (first 14 days after clinical onset) in the Austria (AT) and Italy (IT) survey study cohorts were subjected to two-dimensional multi-dimensional scaling (MDS) with simple matching distance (SMD) between the symptoms. MDS coordinates are presented in point plots. Selected data points are labeled with the symptom names. Percentages of the data set variance associated with the MDS dimensions are indicated in the plot axes. Numbers of complete observations are indicated in the plot captions.

Dim. appetite: diminished appetite; OD: self-reported olfactory dysfunction.

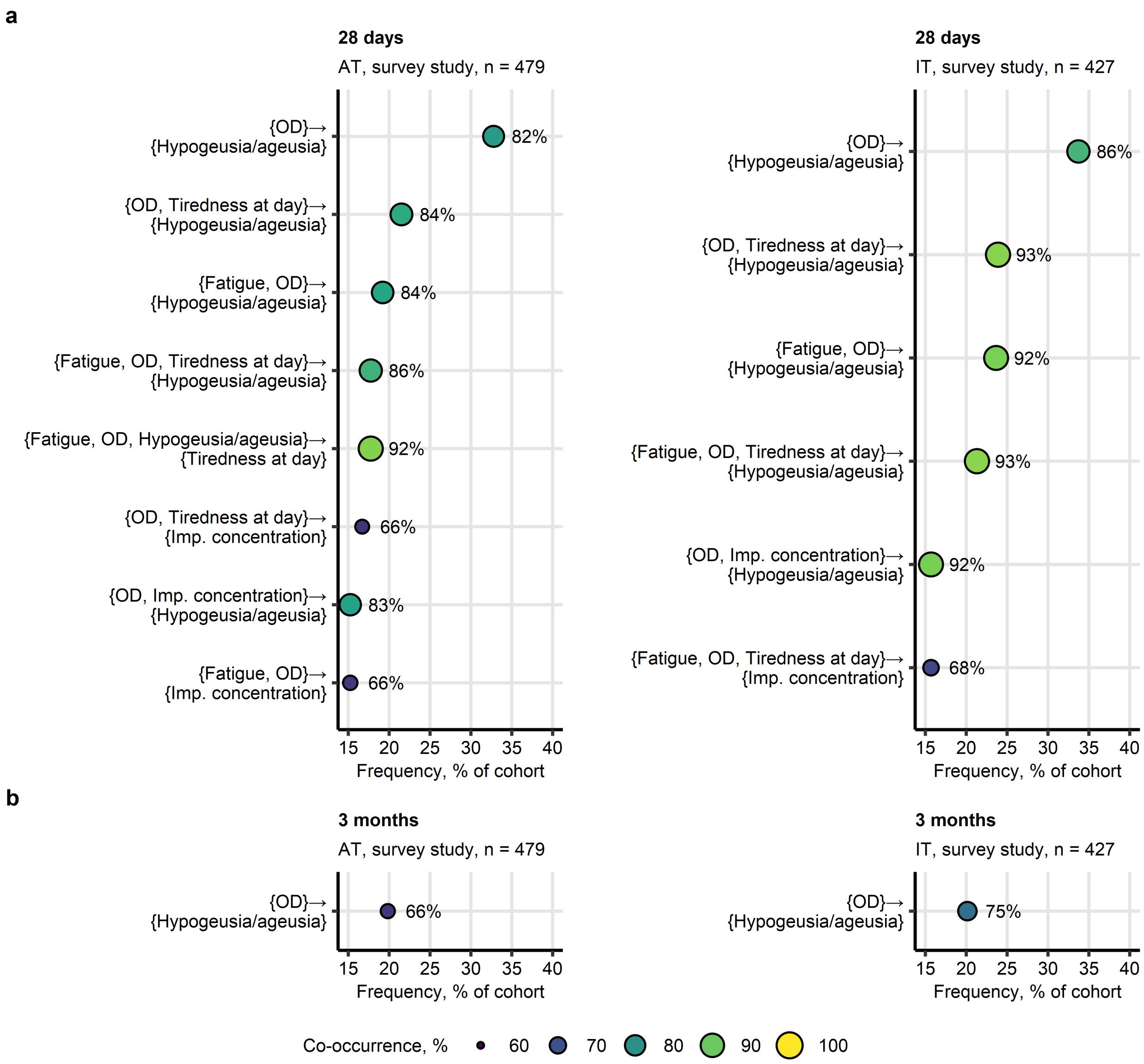

Supplementary Figure S13. Co-occurrence of self-reported olfactory dysfunction and other symptoms in post-acute COVID-19 sequelae.

Frequent combinations (present in >15% of cohort participants) of self-reported olfactory dysfunction (OD) and other symptoms at 28 days (a) and 3 months (b) after clinical onset in the Austria (AT) and Italy (IT) survey study cohorts were identified with the apriori algorithm. Symptom combination frequency and co-occurrence (support statistic) are presented in bubble plots. Point size and color corresponds to co-occurrence, points are labeled with percentages of co-occurrence. Imp. concentration: impaired concentration.

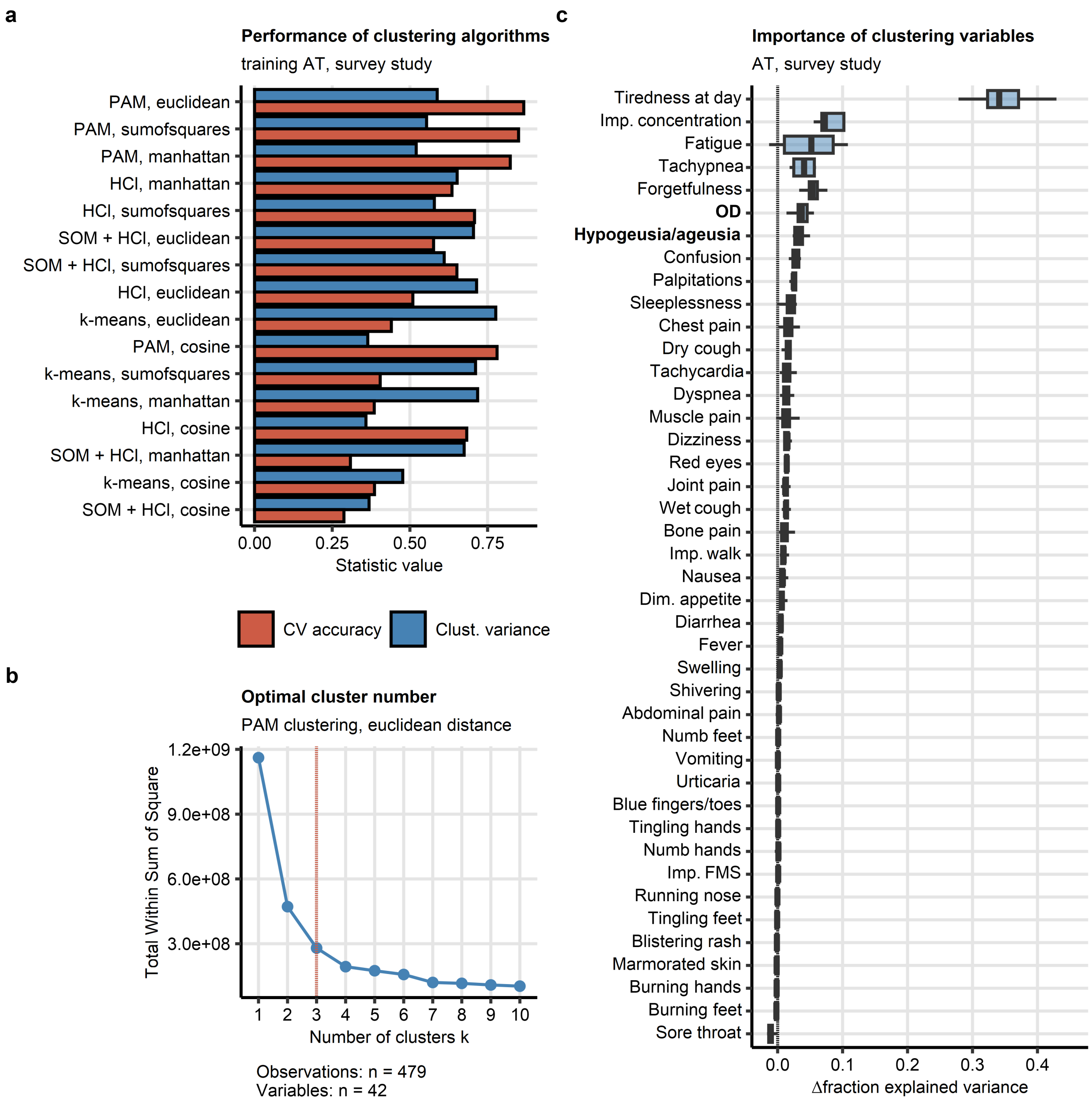

Supplementary Figure S14. Definition of the COVID-19 recovery clusters and clustering feature importance in the survey study.

Individuals of the training Austria (AT) study survey cohort were clustered in respect to symptom-specific recovery times with the PAM (partitioning around medoids) algorithm and Euclidean distance measure.

(a) Comparison of performance of various algorithms (HCl: hierarchical clustering, SOM + HCl: combined self-organizing map and hierarchical clustering, k-means) and distance statistic in clustering of the training data set investigated by clustering variance (ratio of

total between-cluster sum of squares to total sum of squares) and cluster assignment accuracy in 10-fold cross-validation (CV).

(b) Determination of the optimal cluster number in the PAM clustering of the training cohort by the bend of the total within-cluster sum of squares curve.

(c) Permutation importance of the clustering features (symptoms) for clustering of the training cohort expressed as the difference in clustering variance (ratio of total between-cluster sum of squares to total sum of squares) between the initial clustering object and the clustering object with the given variable reshuffled at random. Importance metrics were computed for 20 random permutations of each clustering factor. Median importance metrics with interquartile ranges (IQR) are visualized as boxes, whiskers span over 150% IQR.

OD: self-reported olfactory dysfunction; Dim. appetite: diminished appetite; Imp. concentration: impaired concentration; Imp. walk: impaired walk; Imp. FMS: impaired fine motor skills.

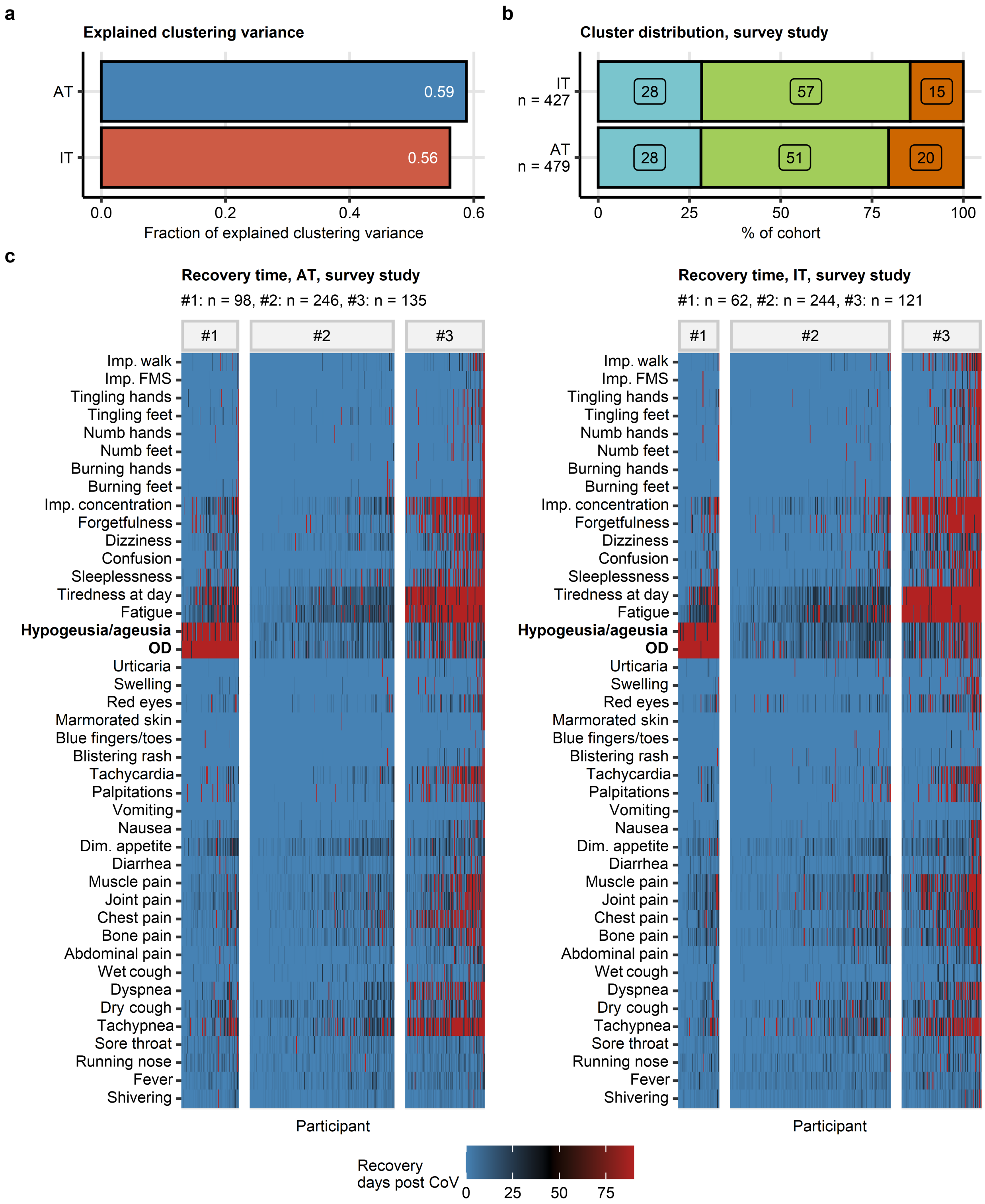
a
Explained clustering variance
AT
IT
0.59
0.56
0.0
0.2
0.4
0.6
Fraction of explained clustering variance
b
Cluster distribution, survey study
IT
n = 427
AT
n = 479
28
57
15
28
51
20
0
25
50
75
100
% of cohort
c
Recovery time, AT, survey study
#1: n = 98, #2: n = 246, #3: n = 135
Recovery time, IT, survey study
#1: n = 62, #2: n = 244, #3: n = 121
#1
#2
#3
Imp. walk
Imp. FMS
Tingling hands
Tingling feet
Numb hands
Numb feet
Burning hands
Burning feet
Imp. concentration
Forgetfulness
Dizziness
Confusion
Sleeplessness
Tiredness at day
Fatigue
Hypogeusia/ageusia
OD
Urticaria
Swelling
Red eyes
Marmorated skin
Blue fingers/toes
Blistering rash
Tachycardia
Palpitations
Vomiting
Nausea
Dim. appetite
Diarrhea
Muscle pain
Joint pain
Chest pain
Bone pain
Abdominal pain
Wet cough
Dyspnea
Dry cough
Tachypnea
Sore throat
Running nose
Fever
Shivering
Participant
Recovery
days post CoV
0
25
50
75

Supplementary Figure S15. Clustering of ambulatory COVID-19 individuals in the survey study by symptom-specific recovery times.

Individuals of the training Austria (AT) survey study cohort were subjected to clustering in respect to symptom-specific recovery times with the PAM (partitioning around medoids) algorithm and Euclidean distance measure (Supplementary Figure S14). Cluster assignment in the test Italy (IT) survey cohort was done with an inverse weighted 7-nearest neighbor (7-NN) classification algorithm.

(a) Fraction of explained clustering variance was computed as a ratio of total between-cluster sum of squares to total sum of squares for the clustering structures in the training AT and test IT cohort. Note similar fractions of explained clustering variances in both cohorts, which suggests good reproducibility of the clustering structure.

(b) Percentages of observations assigned to clusters in the training AT and test IT cohort. Total numbers of complete observations are displayed in the Y axis.

(c) Recovery times for particular COVID-19 symptoms in the COVID-19 recovery clusters presented as heat maps. Numbers of individuals assigned to the recovery clusters are indicated in the plot captions.

OD: self-reported olfactory dysfunction; Dim. appetite: diminished appetite; Imp. concentration: impaired concentration; Imp. walk: impaired walk; Imp. FMS: impaired fine motor skills.

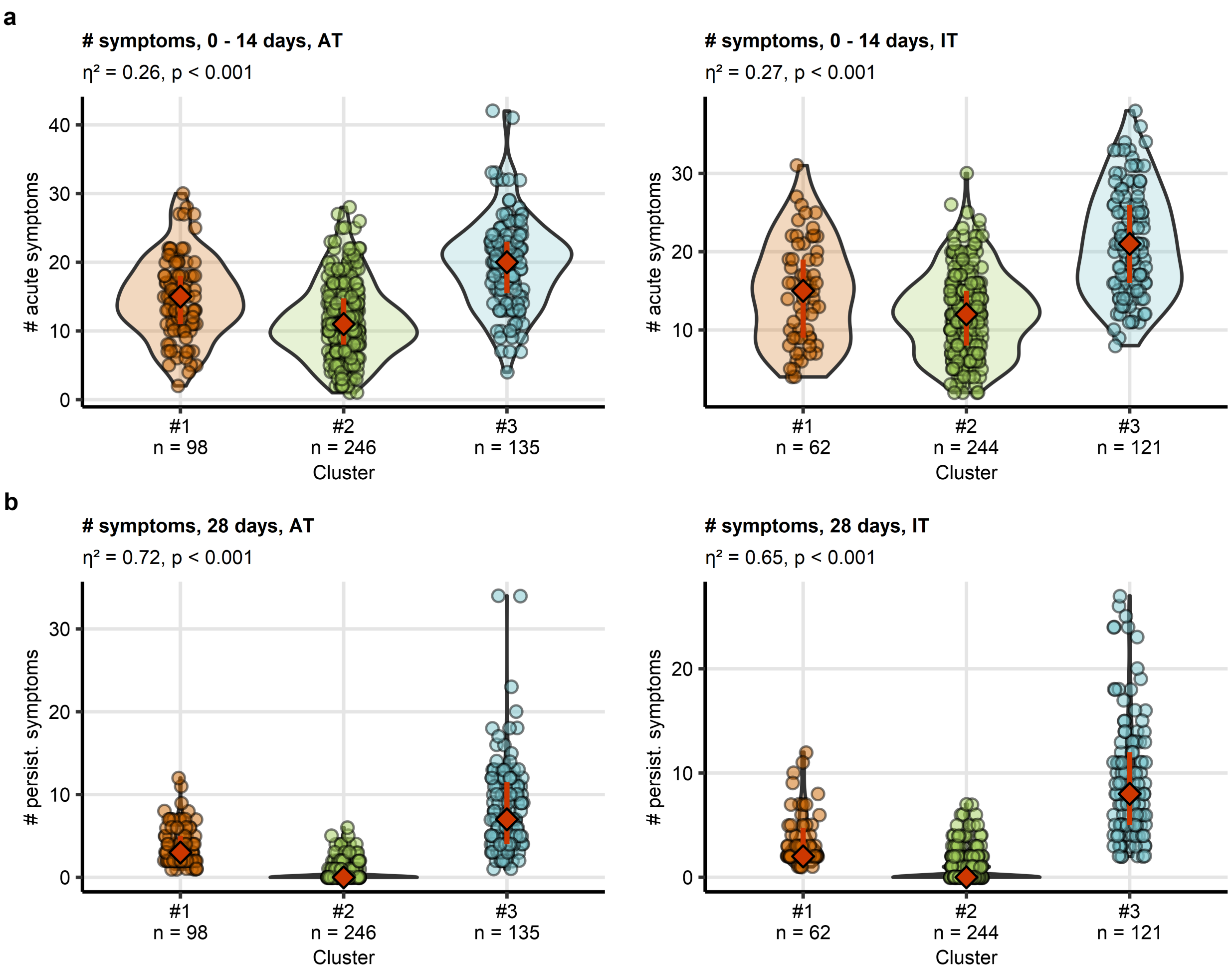

Supplementary Figure S16. Numbers of COVID-19 symptoms in the survey study recovery clusters.

Clustering of the survey study participants in respect to symptom-specific recovery times was done by the semi-supervised PAM algorithm (partitioning around medoids, Euclidean distance, training cohort: Austria [AT], test cohort: Italy [IT]). Differences in numbers of symptoms in the first 14 days (a) and at 28 days (b) after clinical onset between the clusters were assessed by Kruskal-Wallis test and η^2 *effect size statistic. P values were corrected for multiple testing with Benjamini-Hochberg method. Symptom counts are presented in violin plots. Points represent single observations, orange diamonds with whiskers code for medians and interquartile ranges. Effect sizes and p values are indicated in the plot caption. Numbers of complete observations are displayed in the X axes.*

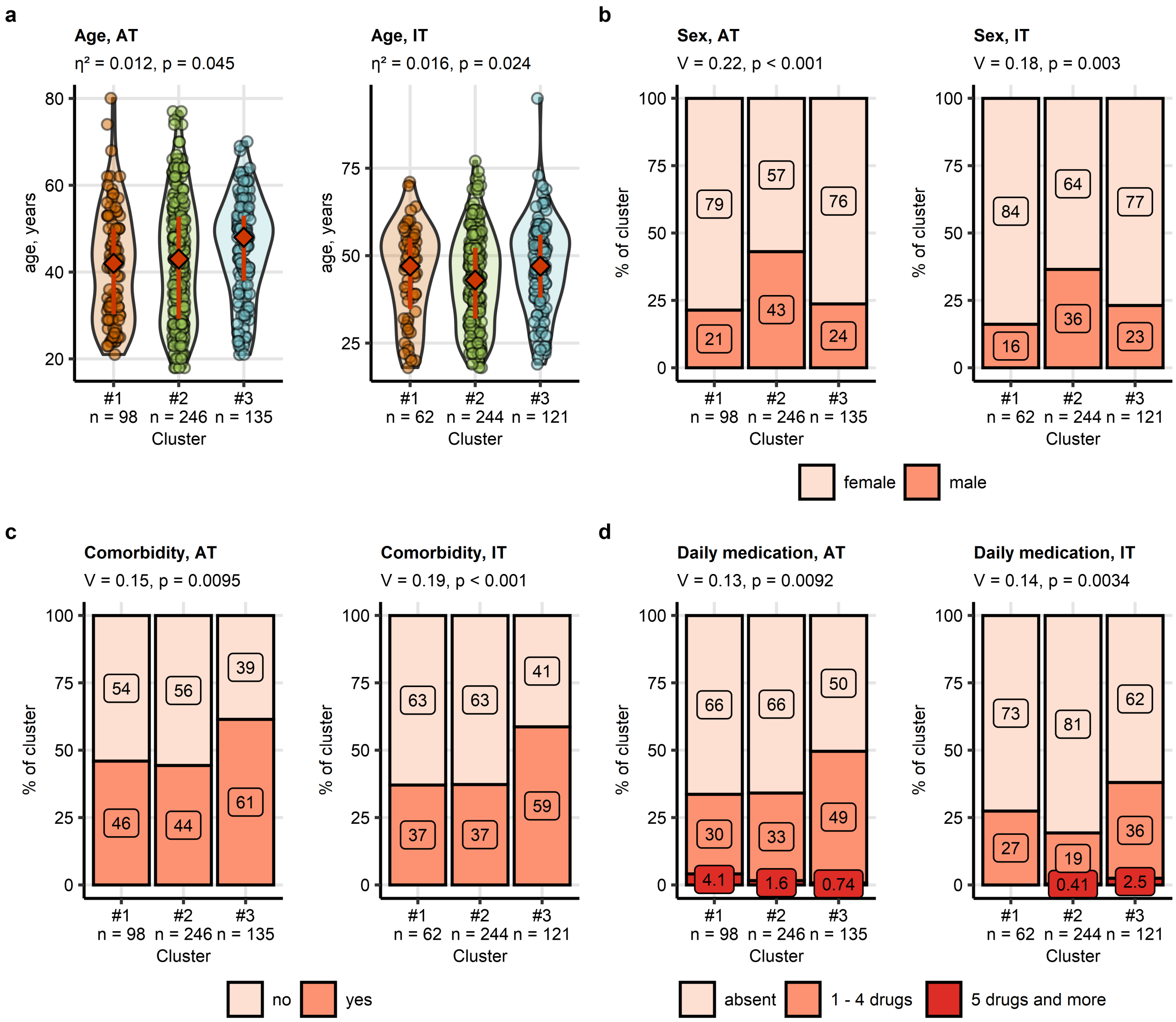

Supplementary Figure S17. COVID-19 recovery clusters of the survey study differ in age, sex distribution, comorbidity and daily medication rates.

Clustering of the survey study participants in respect to symptom-specific recovery times was done by the semi-supervised PAM algorithm (partitioning around medoids, Euclidean distance, training cohort: Austria [AT], test cohort: Italy [IT]). Differences in age (a), sex distribution (b), frequency of comorbidity (c) and daily medication (d) between the recovery clusters were assessed by Kruskal-Wallis test with η^2 *effect size statistic (age) and* χ^2 *test with Cramer V effect size statistic (remaining variables). P values were corrected for multiple testing with Benjamini-Hochberg method. The frequencies are presented as bar plots. Effect sizes and p values are indicated in the plot caption. Numbers of complete observations are displayed in the X axes.*

References

1. Sonnweber T, Sahanic S, Pizzini A, et al (2021) Cardiopulmonary recovery after COVID-19: An observational prospective multicentre trial. European Respiratory Journal 57: https://doi.org/10.1183/13993003.03481-2020

2. Sonnweber T, Tymoszuk P, Sahanic S, et al (2022) Investigating phenotypes of pulmonary COVID-19 recovery: A longitudinal observational prospective multicenter trial. eLife 11: https://doi.org/10.7554/ELIFE.72500

3. Hüfner K, Tymoszuk P, Ausserhofer D, et al (2022) Who Is at Risk of Poor Mental Health Following Coronavirus Disease-19 Outpatient Management? Frontiers in Medicine 9: https://doi.org/10.3389/fmed.2022.792881

4. Sahanic S, Tymoszuk P, Ausserhofer D, et al (2022) Phenotyping of Acute and Persistent Coronavirus Disease 2019 Features in the Outpatient Setting: Exploratory Analysis of an International Cross-sectional Online Survey. Clinical infectious diseases : an official publication of the Infectious Diseases Society of America 75:e418–e431. https://doi.org/10.1093/cid/ciab978

5. Löwe B, Wahl I, Rose M, et al (2010) A 4-item measure of depression and anxiety: Validation and standardization of the Patient Health Questionnaire-4 (PHQ-4) in the general population. Journal of Affective Disorders 122:86–95. https://doi.org/10.1016/j.jad.2009.06.019

6. Gräfe K, Zipfel S, Herzog W, Löwe B (2004) Screening psychischer störungen mit dem "Gesundheitsfragebogen für Patienten (PHQ-D)". Ergebnisse der Deutschen validierungsstudie. Diagnostica 50:171–181. https://doi.org/10.1026/0012-1924.50.4.171

7. Rass V, Beer R, Schiefecker AJ, et al (2021) Neurological outcome and quality of life 3 months after COVID-19: A prospective observational cohort study. European Journal of Neurology 28:3348–3359. https://doi.org/10.1111/ene.14803

8. Whitcroft KL, Merkonidis C, Cuevas M, et al (2016) Intranasal sodium citrate solution improves olfaction in post-viral hyposmia. Rhinology 54:368–373. https://doi.org/10.4193/RHINO16.054

9. Masala C, Cavazzana A, Sanna F, et al (2022) Correlation between olfactory function, age, sex, and cognitive reserve index in the Italian population. European Archives of Oto-Rhino-Laryngology 279:4943. https://doi.org/10.1007/S00405-022-07311-Z

10. Damm M, Pikart LK, Reimann H, et al (2014) Olfactory training is helpful in postinfectious olfactory loss: a randomized, controlled, multicenter study. The Laryngoscope 124:826–831. https://doi.org/10.1002/LARY.24340

11. Hummel T, Kobal G, Gudziol H, Mackay-Sim A (2007) Normative data for the "Sniffin' Sticks" including tests of odor identification, odor discrimination, and olfactory thresholds: an upgrade based on a group of more than 3,000 subjects. European archives of oto-rhino-laryngology : official journal of the European Federation of Oto-Rhino-Laryngological Societies (EUFOS) : affiliated with the German Society for Oto-Rhino-Laryngology - Head and Neck Surgery 264:237–243. https://doi.org/10.1007/S00405-006-0173-0

12. R Core Team, Bivand R, Carey VJ, et al (2022) foreign: Read Data Stored by 'Minitab', 'S', 'SAS', 'SPSS', 'Stata', 'Systat', 'Weka', 'dBase', ...

13. Wickham H, Bryan J, Posit P, et al (2022) readxl: Read Excel Files

14. Wickham H, Averick M, Bryan J, et al (2019) Welcome to the Tidyverse. Journal of Open Source Software 4:1686. https://doi.org/10.21105/joss.01686

15. Henry L, Wickham Hadley (2022) rlang: Functions for Base Types and Core R and 'Tidyverse' Features

16. Gagolewski M, Tartanus B (2021) Package 'stringi'

17. Kassambara A (2021) rstatix: Pipe-Friendly Framework for Basic Statistical Tests

18. Meyer D, Zeileis A, Hornik K (2021) vcd: Visualizing Categorical Data

19. Mangiafico S (2022) rcompanion: Functions to Support Extension Education Program Evaluation

20. Bates D, Mächler M, Bolker BM, Walker SC (2015) Fitting linear mixed-effects models using lme4. Journal of Statistical Software 67:1–48. https://doi.org/10.18637/jss.v067.i01

21. Kuznetsova A, Brockhoff PB, Christensen RHB (2017) lmerTest Package: Tests in Linear Mixed Effects Models. Journal of Statistical Software 82:1–26. https://doi.org/10.18637/JSS.V082.I13

22. Hahsler M, Grün B, Hornik K (2005) Arules - A computational environment for mining association rules and frequent item sets. Journal of Statistical Software 14: https://doi.org/10.18637/JSS.V014.I15

23. Schubert E, Rousseeuw PJ (2019) Faster k-Medoids Clustering: Improving the PAM, CLARA, and CLARANS Algorithms. In: Lecture notes in computer science (including subseries lecture notes in artificial intelligence and lecture notes in bioinformatics). Springer, pp 171–187

24. Drost H-G (2018) Philentropy: Information Theory and Distance Quantification with R. Journal of Open Source Software 3:765. https://doi.org/10.21105/joss.00765

25. Schwender H, Fritsch A (2018) scrime: Analysis of High-Dimensional Categorical Data Such as SNP Data

26. Kassambara A, Mundt F (2020) factoextra: Extract and Visualize the Results of Multivariate Data Analyses

27. Kuhn M (2008) Building predictive models in R using the caret package. Journal of Statistical Software 28:1–26. https://doi.org/10.18637/jss.v028.i05

28. Vaughan D, Dancho M, RStudio (2022) furrr: Apply Mapping Functions in Parallel using Futures

29. Wickham Hadley (2016) ggplot2: Elegant Graphics for Data Analysis, 1st ed. Springer-Verlag, New York

30. Sachs MC (2017) Plotroc: A tool for plotting ROC curves. Journal of Statistical Software 79:1–19. https://doi.org/10.18637/jss.v079.c02

31. Wilke CO, Wiernik BM (2022) ggtext: Improved Text Rendering Support for 'ggplot2'

32. Wilke CO (2019) Fundamentals of Data Visualization: A Primer on Making Informative and Compelling Figures, 1st ed. O'Reilly Media, Sebastopol

33. Gohel D (2022) flextable: Functions for Tabular Reporting

34. Allaire J, Xie Y, McPherson J, et al (2022) rmarkdown: Dynamic Documents for R

35. Xie Y (2022) knitr: A General-Purpose Package for Dynamic Report Generation in R

36. Xie Y (2016) Bookdown: Authoring books and technical documents with R Markdown

37. Sahanic S, Tymoszuk P, Luger AK, et al (2023) COVID-19 and its continuing burden after 12 months: a longitudinal observational prospective multicentre trial. ERJ open research 9:00317–2022. https://doi.org/10.1183/23120541.00317-2022

38. Cohen J (2013) Statistical Power Analysis for the Behavioral Sciences. Statistical Power Analysis for the Behavioral Sciences. https://doi.org/10.4324/9780203771587

39. McHugh ML (2012) Interrater reliability: the kappa statistic. Biochemia Medica 22:276. https://doi.org/10.11613/bm.2012.031

40. Serlin RC, Carr J, Marascuilo LA (1982) A measure of association for selected nonparametric procedures. Psychological Bulletin 92:786–790. https://doi.org/10.1037/0033-2909.92.3.786

41. Fleiss JL, Cohen J, Everitt BS (1969) Large sample standard errors of kappa and weighted kappa. Psychological Bulletin 72:323–327. https://doi.org/10.1037/h0028106

42. Benjamini Y, Hochberg Y (1995) Controlling the False Discovery Rate: A Practical and Powerful Approach to Multiple Testing. Journal of the Royal Statistical Society: Series B (Methodological) 57:289–300. https://doi.org/10.1111/j.2517-6161.1995.tb02031.x

43. Boriah S, Chandola V, Kumar V (2008) Similarity measures for categorical data: A comparative evaluation. In: Society for industrial and applied mathematics - 8th SIAM international conference on data mining 2008, proceedings in applied mathematics 130. pp 243–254

44. Agrawal R, Imieliński T, Swami A (1993) Mining association rules between sets of items in large databases. ACM SIGMOD Record 22:207–216. https://doi.org/10.1145/170036.170072

45. Lange T, Roth V, Braun ML, Buhmann JM (2004) Stability-based validation of clustering solutions. Neural Computation 16:1299–1323. https://doi.org/10.1162/089976604773717621

46. Leng M, Wang J, Cheng J, et al (2014) Adaptive semi-supervised clustering algorithm with label propagation. Journal of Software Engineering 8:14–22. https://doi.org/10.3923/jse.2014.14.22
